# A transient ischemic environment induces reversible compaction of chromatin

**DOI:** 10.1186/s13059-015-0802-2

**Published:** 2015-11-05

**Authors:** Ina Kirmes, Aleksander Szczurek, Kirti Prakash, Iryna Charapitsa, Christina Heiser, Michael Musheev, Florian Schock, Karolina Fornalczyk, Dongyu Ma, Udo Birk, Christoph Cremer, George Reid

**Affiliations:** Institute for Molecular Biology, 55128 Mainz, Germany; Institute of Pharmacy and Molecular Biotechnology, University of Heidelberg, 69120 Heidelberg, Germany; Department of Molecular Biophysics, University of Łódź, Łódź, Poland; Centre for Biomedicine and Medical Technology Mannheim (CBTM), University of Heidelberg, 68167 Mannheim, Germany

**Keywords:** Myocardial cell, Cardiomyocyte, Ischemia, Hypoxia, Nutrient deprivation, Chromatin, Chromatin condensation, ATP, Mg^2+^, Polyamines, Spermine, Spermidine, Nucleosome, Super-resolution, Microscopy, FRAP, DNAseI, Single Molecule Localization Microscopy, Cytometry, Side-scatter

## Abstract

**Background:**

Cells detect and adapt to hypoxic and nutritional stress through immediate transcriptional, translational and metabolic responses. The environmental effects of ischemia on chromatin nanostructure were investigated using single molecule localization microscopy of DNA binding dyes and of acetylated histones, by the sensitivity of chromatin to digestion with DNAseI, and by fluorescence recovery after photobleaching (FRAP) of core and linker histones.

**Results:**

Short-term oxygen and nutrient deprivation of the cardiomyocyte cell line HL-1 induces a previously undescribed chromatin architecture, consisting of large, chromatin-sparse voids interspersed between DNA-dense hollow helicoid structures 40–700 nm in dimension. The chromatin compaction is reversible, and upon restitution of normoxia and nutrients, chromatin transiently adopts a more open structure than in untreated cells. The compacted state of chromatin reduces transcription, while the open chromatin structure induced upon recovery provokes a transitory increase in transcription. Digestion of chromatin with DNAseI confirms that oxygen and nutrient deprivation induces compaction of chromatin. Chromatin compaction is associated with depletion of ATP and redistribution of the polyamine pool into the nucleus. FRAP demonstrates that core histones are not displaced from compacted chromatin; however, the mobility of linker histone H1 is considerably reduced, to an extent that far exceeds the difference in histone H1 mobility between heterochromatin and euchromatin.

**Conclusions:**

These studies exemplify the dynamic capacity of chromatin architecture to physically respond to environmental conditions, directly link cellular energy status to chromatin compaction and provide insight into the effect ischemia has on the nuclear architecture of cells.

**Electronic supplementary material:**

The online version of this article (doi:10.1186/s13059-015-0802-2) contains supplementary material, which is available to authorized users.

## Background

Cellular oxygen insufficiency, hypoxia, occurs in physiological and developmental processes and in disease, such as in solid tumors, stroke and cardiac infarction. Hypoxia in pathological situations often results from ischemia and is associated with a concomitant reduced availability of glucose. The major transcriptional mediator in hypoxia is the alpha/beta-heterodimeric hypoxia-inducible factor (HIF), which persists in cells only when intracellular oxygen is low [1]. HIF activates the expression of genes involved in oxygen transport, glucose uptake, glycolysis and angiogenesis [2, 3]. Additionally, ischemia-induced hypoglycemia results in stimulation of AMP-activated protein kinase (AMPK), a stress sensor which induces catabolic pathways and down-regulates anabolic processes such as fatty acid oxidation, glucose uptake and glycolysis upon cellular energy insufficiency [4, 5]. Moderate periods of hypoxia and nutrient deprivation provoke a predominant global repression of transcription [6, 7], although activation of hypoxia and/or hypoglycemic responsive genes does occur within this general transcriptionally repressive environment [8]. The transcriptional competence of DNA in eukaryotic cells is determined by its organization in chromatin. Chromatin structure is dynamically regulated at multiple levels, which include ATP-dependent chromatin remodeling [9], post-translational modification of chromatin [10] and the incorporation of histone variants [10].

The metabolic status of cells has a direct effect upon chromatin architecture, as many histone-modifying enzymes utilize essential metabolites, such as ATP, NAD^+^, acetyl-coenzyme A (acetyl-CoA), S-adenosylmethionine or oxygen, either as cofactors or as substrates [11]. In particular, histone acetylation is dependent upon the action of ATP-citrate lyase [12], which converts mitochondrially derived citrate into cytoplasmically available acetyl-CoA. Additionally, molecular oxygen is required as a substrate by the Jumonji C (JmjC) class of dioxygenases to achieve histone demethylation. Consequently, hypoxia can limit the activity of a subset of JmjC histone demethylases, resulting in global increases in the methylation of histone H3K4, H3K9, H3K27 and H3K36 and in chromatin condensation [13]. Moreover, moderate hypoxia is reported to induce an overall decrease of H3K9 acetylation [14], with ischemia shown to decrease histone H4K16 acetylation in neural cells [15].

Nuclear architecture is dynamic and represents the structural and topological product of epigenetic regulation (for reviews see [16–18]). Chromosomes occupy distinct territories in the cell nucleus [18–20], which harbor chromatin domains (CDs) with a size range in the order of 100 kbp to 1 Mbp [21–23]. In turn, CDs form chromatin domain clusters (CDCs) with a compact core and a less compact periphery, known as the perichromatin region [24–26]. Histone marks associated with transcriptionally silent chromatin are enriched in the interior of CDCs, whereas marks typical for transcriptionally competent chromatin, and for chromatin associated with transcribing RNA polymerase II, are enriched in the perichromatin region, where nascent RNA is synthesized [25–28]. CDCs, in turn, form a higher order chromatin network, which is attached to the nuclear envelope and permeates the nuclear interior. This chromatin network is co-aligned with a second network, called the interchromatin compartment, which starts at nuclear pores [25, 29]. It pervades the nuclear space between CDCs and is enriched in proteins involved in genomic output. Previous work has demonstrated that chromatin architecture physically responds to environmental conditions, with condensation occurring in response to hyperosmotic conditions [30] and in response to oxidative stress provoked by the fungal metabolite chaetocin [31]. Depletion of ATP in HeLa cells results in chromatin compaction as evaluated by fluorescence lifetime imaging microscopy-Förster resonance energy transfer (FLIM-FRET) [32]. Reflecting this, stress-induced and developmentally induced changes of gene expression correspond with major changes of nuclear organization [33]. As ischemia provokes major changes in transcriptional output and the post-translational modification of histones and a reduction in intracellular ATP levels, it can be anticipated that oxygen and nutrient deprivation (OND) may result in significant changes to nuclear architecture.

While various biochemical approaches exist to evaluate the compaction state of chromatin — for example, chromatin capture technology [34] — they do not report the underlying three-dimensional nuclear structure. Recent advances in super-resolution optical microscopy provide structural discrimination comparable to that of electron microscopy [35]. Currently, single molecule localization microscopy (SMLM) has the highest spatial resolution of all optical microscopic methods used in cellular nanostructure analysis [36]. In the SMLM mode used here [37], most fluorophores are transferred to a metastable dark state, while a minor population of multiple emitting fluorophores remains that are optically isolated and, hence, can be individually localized. In a typical SMLM determination, tens of thousands of frames are acquired over a period of several hours. Integrating the positions of fluorophores results in a joint localization map that can resolve spatial features in the order of 30–100 nm, in comparison with the approximately 250 nm limit of conventional optical methods [38, 39]. Direct imaging of DNA by means of localization microscopy is a prerequisite to determining chromatin structure, and has recently been accomplished for a range of DNA binding dyes [37, 40–43].

We describe optically, at single molecule resolution, the effect of the consequences of ischemia on the nuclear architecture of immortalized cardiomyocytes. Exposure of HL-1 cells, an adult murine cardiomyocyte cell line [44], to moderate, acute hypoxia (1 % O_2_ for 1 hour), combined with nutrient starvation and glycolytic blockade, induces a condensed, hollow, whorl-like chromatin configuration with a concomitant reduction (around 30 %) in the capacity of chromatin to associate with the DNA selective dye Vybrant DyeCycle Violet. Significantly, the occurrence of decondensed chromatin, as characterized by a diffuse distribution of DNA at the edge of chromatin territories, marked by the local presence of acetylated histones, is ablated. Condensed chromatin exhibits an increased resistance to digestion with DNAseI compared with chromatin in untreated cells and, additionally, the mobility of linker histone H1, as estimated by fluorescent recovery after photobleaching (FRAP), is significantly reduced by OND. Relaxation of nuclear architecture occurs within tens of minutes upon cessation of OND. Cytometric analysis of immunostained cells confirms and extends the findings made by SMLM studies. Mechanistically, chromatin compaction is associated with depletion of the intracellular pool of ATP, which results in the relocation of a substantial proportion of the cellular polyamine pool from the cytoplasm to the nucleus.

## Results

### OND of HL-1 cells induces chromatin compaction

**Fig. 1:**
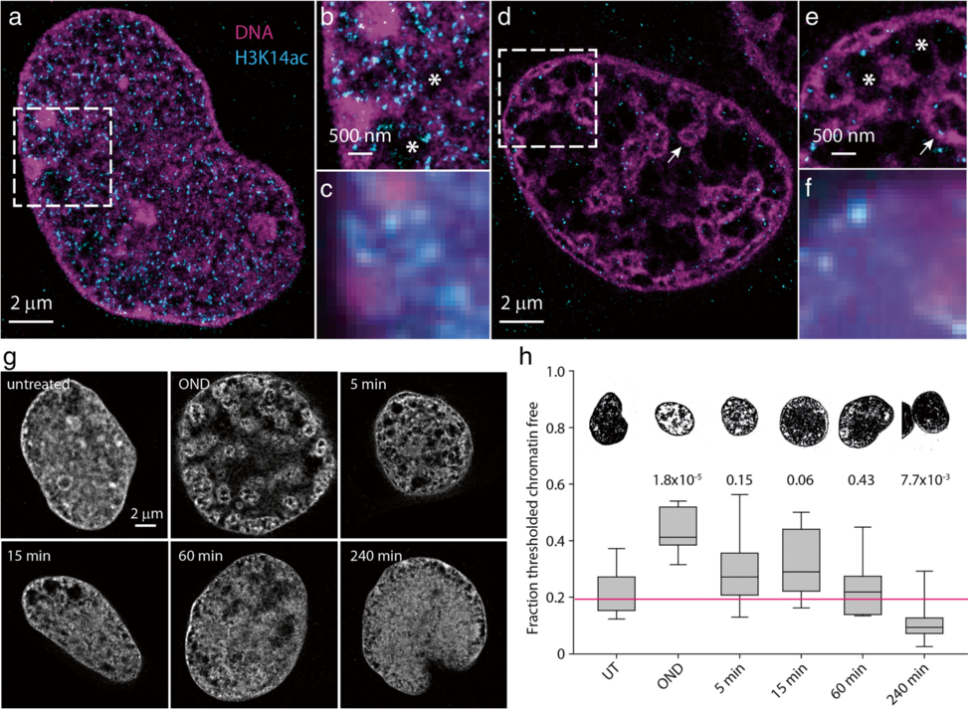
Oxygen and nutrient deprivation induces compaction of chromatin. HL-1 cells were fixed, permeabilized, and immunostained with anti-acetylated histone H3K14 and then counter-stained with Vybrant DyeCycle Violet. Two-color SMLM was performed on untreated HL-1 cells (**a**, **b**) or on cells exposed to 1 hour of OND (**d**, **e**). The *dashed boxes* in (**a**, **d**) are shown as zoomed views in (**b**) and (**e**), respectively. For comparison, wide-field images of the inset regions are shown in (**c**, **f**). Chromatin voids are indicated by *asterisks* and atolls marked by the *arrow*. Representative SMLM images of Vybrant Dyecycle Violet-stained nuclei, either untreated, subjected to 1 hour of OND or 5, 15, 60 and 240 minutes after release from OND are shown in (**g**). A discriminatory threshold (pixel intensity ≤ 50) was applied to the experimental set of SMLM imaged nuclei (a minimum of nine cells were imaged), with box plots and representative images describing the median and range of the proportion of the nucleus with chromatin shown in (**h**). *P* values compared with untreated are reported above the box plots. *UT* untreated

We firstly assessed the response of chromatin to experimental conditions that mimic ischemia-reperfusion using a two-color SMLM to characterize the response and recovery of nuclear architecture to transient OND in HL-1 cells, as evaluated by staining of DNA with DNA binding dyes and by immunodetection of H3K14ac, a histone mark associated with transcriptionally permissive chromatin. Fixed and permeablized cells were immunostained using AlexaFluor 647 conjugated anti-H3K14ac and counter-stained with Vybrant DyeCycle Violet, a photoconvertible DNA binding dye which undergoes reversible photoswitching and can be utilized for SMLM based on blinking, with individual fluorophores emitting up to 1500 photons per cycle [43]. We typically generated localization maps, for at least nine nuclei per experimental condition, by integrating 30,000 observations, each of which captured photons emitted during a 50 ms exposure period. These observations localize individual fluorophores at sub-diffractional precision, with a theoretical lateral optical resolution of 67 nm and an experimentally determined structural resolution of 100 nm. A short movie illustrating how localization maps were generated for DNA binding dyes is presented in Additional file 1. These values are less than the typical spatial resolution achieved when imaging surface structures such as membrane bound proteins, where typical values of spatial resolution are in the range of 20 nm. This occurs as a consequence of imaging inside optically inhomogeneous media, such as when focusing through several layers of membranes and organelles into the cell nucleus. We discuss these limitations extensively in Notes N1 and N2 in Additional file 2. As shown in Fig. 1a, b, untreated HL-1 cells show a typical DNA staining pattern, with rather intense staining occurring just inside the nuclear envelope and in discrete foci within the nucleus. There is a general diffuse staining of DNA within the nucleus, with small inter-nuclear compartments clearly visible between individual chromatin domains. H3K14ac occurs in a punctate distribution throughout the nucleus, with individual foci predominantly located at the edge of chromatin domains. This is in keeping with the topography found for the transcriptionally permissive H3K4me3 modification in a range of mammalian cell types [25, 26]. The level of resolution and precision of localization obtained with our dual-color SMLM technique cannot be attained by conventional microscopy (Fig. 1c).

SMLM imaging of the nuclei of HL-1 cells exposed to 1 hour of OND demonstrates that an ischemic environment provokes a dramatic change in nuclear architecture, with condensed chromatin present at the subnuclear envelope, often as a closely spaced double arrangement of densely stained DNA or as hollow intranuclear atolls (Fig. 1d, e). Moreover, the interchromosomal space consists of large, DNA-sparse voids, with little of the diffuse DNA staining that is seen in untreated cells. Given that OND induced toroidal structures, we investigated if these arose as a consequence of invagination of the nuclear envelope or through perturbation of the distribution of lamin. OND does not promote invagination of the nuclear envelope (Figure S1 in Additional file 2) or change the structural distribution of lamin B1 (Figure S2 in Additional file 2).

A decrease in staining for H3K14ac occurs upon OND, with SMLM imaging again demonstrating that the remaining H3K14ac occurs largely at the edge of chromatin domains. In order to experimentally evaluate the effects of reperfusion following a transient ischemic period, we next evaluated the response of OND-induced chromatin compaction to the restitution of normoxia and nutrients. SMLM images of representative HL-1 cells either untreated, subjected to 1 hour of OND or upon subsequent recovery from OND are shown in Fig. 1g. Following OND-induced chromatin compaction, nuclear architecture relaxes and at 4 hours post-OND acquires a more open conformation than in untreated cells. To quantitatively evaluate this, we applied a discriminatory threshold to the experimental set of SMLM imaged cells to delimit chromatin-sparse nuclear regions. The distribution of nuclear areas that are chromatin-sparse is reported in Fig. 1h, with representative thresholded images shown above. OND induces an approximately twofold increase in chromatin-free nuclear area. Sixty minutes of recovery from OND is sufficient for the majority of cells to restore chromatin architecture; however, a significant proportion of cells adopt a more open chromatin structure at 240 minutes. HL-1 cells recover fully from transient OND and continue to proliferate as well as untreated cells.

### Alternative staining and SMLM methodologies confirm that OND induces chromatin compaction

**Fig. 2:**
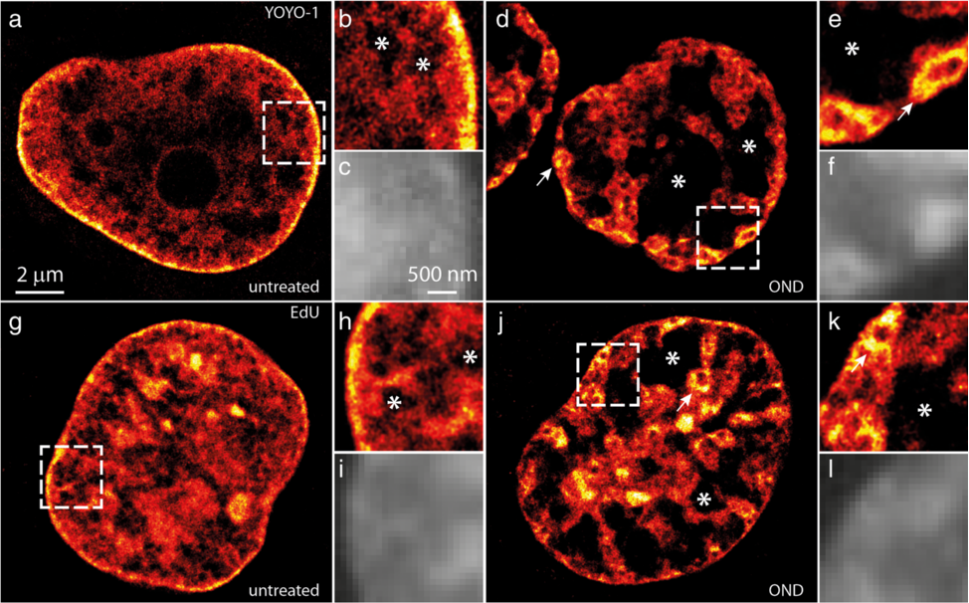
Alternative dyes and labeling methodologies confirm OND-induced compaction of chromatin. HL-1 cells, either untreated (**a**–**c**) or exposed to 1 hour of OND (**d**–**f**) were fixed, permeabilized, stained with the DNA binding dye YOYO-1 and subjected to SMLM (**a**, **b**, **d**, **e**). Alternatively, cells were labeled for 24 hours with 10 μM 5-ethynyl-2′-deoxyuridine (EdU), and then were either untreated (**g**–**i**) or subjected to 1 hour of OND (**j**–**l**). Following fixation, EdU incorporated into DNA was coupled via click chemistry to AlexaFluor 488, as described [46], and nuclear DNA determined by SMLM (**g**, **h**, **j**, **k**). The *dashed boxes* in (**a**, **d**, **g**, **j**) are shown as zoomed views in (**b**), (**e**), (**h**) and (**k**), respectively. For comparison, wide-field images of the inset regions are shown in (**c**, **f**, **i**, **l**). Chromatin voids are indicated by an *asterisk*, with atolls marked by an *arrow*

We then confirmed that OND induces extensive compaction of chromatin using an alternative nucleic acid binding dye, YOYO-1 [45], that also blinks under our experimental conditions, as previously reported [40] (Fig. 2a–f) and with a click-chemistry approach chemically linking a fluorophore to 5-ethynyl-2′-deoxyuridine (EdU) [46] incorporated into DNA during cellular replication (Fig. 2g–l). While the density of signals is reduced in comparison to Vybrant DyeCycle Violet with both these approaches, they clearly demonstrate that 1 hour of OND induces chromatin compaction in HL-1 cardiomyocytes. A presentation on why binding activated localization microscopy (BALM) is not suitable for imaging YOYO-1 in mammalian cell nuclei is shown in Figure S3 in Additional file 2. Additionally, we evaluated OND-induced chromatin compaction using structured illumination microscopy (SIM). In contrast to untreated cells (Figure S4 in Additional file 2), OND induces large DNA-free voids in nuclei (Figure S5 in Additional file 2).

### A quantitative binning analysis describes the extent of chromatin compaction, the range of sizes of condensed structures and illustrates that chromatin adopts a more open structure on recovery from OND

**Fig. 3:**
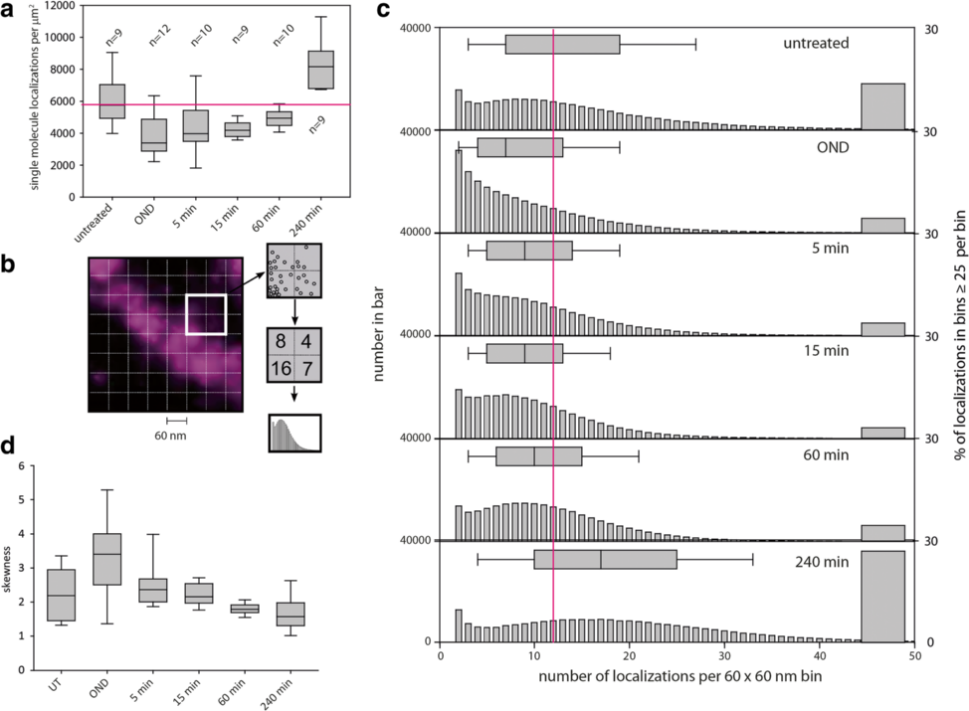
Quantification of chromatin compaction through binning. The influence of OND on the nuclear distribution and accessibility of chromatin was characterized by analysis of joint localization maps generated by SMLM. **a** The median and range of densities of single molecule localizations, calculated over the entire nucleus and for a minimum of nine cells, for untreated, OND exposed and recovering cells. A binning approach, outlined in (**b**), was then used to characterize the extent of chromatin compaction as cells transition from normal conditions through OND and in recovery from OND (**c**), with the median and range of the distribution shown above each histogram. The proportion of bins containing ≥ 25 localizations is presented as a *bar* on the right of each panel. **d** As the distributions of histograms of binned data differ significantly between time points, the skewness (deviation from the mean) was calculated for all of the images. *UT* untreated

SMLM defines the spatial localization of single fluorophores, permitting a quantitative assessment of chromatin condensation induced by OND. We initially evaluated the density of Vybrant DyeCycle Violet molecules detected by SMLM (Fig. 3a). Untreated cells have a median value of around 6 × 10^3^ dye localizations per μm^2^, which decreases by approximately 30 % upon 1 hour of OND, and then recovers upon release from ischemia-mimetic conditions. Significantly, chromatin associates with approximately 30 % more Vybrant DyeCycle Violet 4 hours after release from OND compared with untreated cells, again suggesting that chromatin adopts, at least transiently, a more open configuration upon recovery from ischemic conditions. Moreover, in contrast to the generally open structure of chromatin in untreated cells, OND induces an average thickness of chromatin structures of 120 nm (Figure S6 in Additional file 2), which is confirmed by Fourier radial correlation analysis (Figure S7 in Additional file 2), where an average chromatin thickness of 130 nm is obtained.

We then used a binning approach to quantify the subnuclear distribution of chromatin, through counting individual SMLM locations of individual Vybrant Violet-labeled DNA sites within a grid of squares (bins) overlaid upon the image of the nucleus (Fig. 3b). Reflecting the development of extensive regions within the nucleus that become chromatin-sparse upon OND, the number of molecules present per bin becomes reduced in experimental ischemia, and this recovers upon restoration of normoxia and nutrients (Fig. 3c). A bin size of 60 × 60 nm was chosen to illustrate that this technique resolves structures at the tens of nanometers scale, with bins containing either zero or one localization excluded from the presented results. Reflecting the increase in DNA dye binding seen 4 hours post-OND, there is a corresponding increase at 4 hours in the proportion of bins containing a large number of localizations (Fig. 3c), indicating that recovery from OND induces chromatin to adopt, at least transiently, a more open conformation. In order to describe the spatial extent of changes of chromatin density induced by OND, we evaluated a range of bin sizes of between 10 and 500 nm for untreated and OND cells. We then evaluated the skewness, a measure of asymmetry around the mean, of the distributions throughout the experimental time-course (Fig. 3d) and found that the distribution of chromatin density became more skewed in the direction of high DNA density classes than in untreated cells (median skewness of ~3.2 and ~2.2, respectively). Notably, as the skewness parameter is positive in all experimental conditions, including untreated cells, it can be inferred that the majority of chromatin is located within a highly condensed state rather than in a diffuse conformation. Similar results were obtained for EdU-Alexa 488-labeled chromatin (Figure S8 in Additional file 2).

### Nearest neighbor analysis confirms and describes the extent of chromatin compaction

**Fig. 4:**
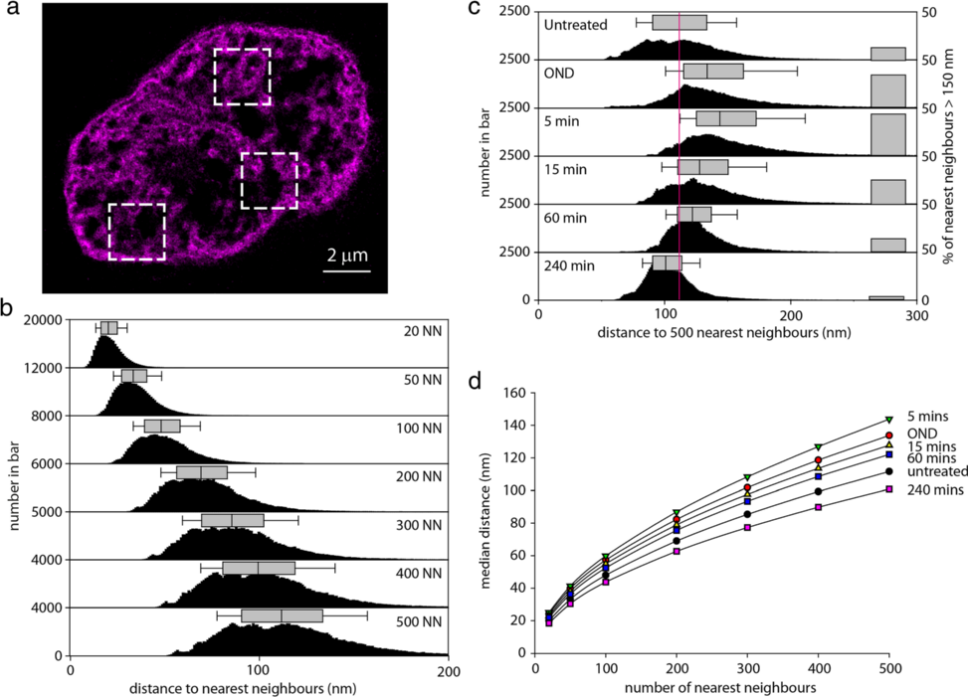
Nearest neighbor characterization of OND-induced chromatin compaction. Nearest neighbor analysis was used to describe the extent of chromatin compaction upon deprivation of oxygen and nutrients using three internal regions of interest (ROIs; outlined with *dashed boxes*), as illustrated for an HL-1 cell subjected to 1 hour of OND (**a**). Results were generated for each experimental condition using three ROIs per nucleus and three nuclei per determination. **b** The effect of the number of nearest neighbors evaluated on the distance to the analyzed position is shown as a histogram and as a box plot showing the median and range of distribution of values for untreated cells. **c** The extent of chromatin compaction as cells transition from normal conditions through OND and in recovery from OND, using the distance to 500 nearest neighbors, with the median and range of distribution shown above each histogram. The proportion of bins with a distance to 100 nearest neighbors ≥ 80 nm is shown as a bar on the right of each panel. **d** The relationship between the number of nearest neighbors used in the analysis to the median distance to the set of nearest neighbors for each experimental condition

We further characterized chromatin condensation induced by OND by determining the average distance of single molecule localizations to variable numbers of nearest neighbors within computationally tractable representative regions of interest (ROIs). An example of selected ROIs is shown in Fig. 4a. Three ROIs from three independent nuclei were used to generate datasets for each experimental condition. We first evaluated the relationship between the average distance to nearest neighbors and the number of neighbors evaluated. The median distance and range of distribution rises with the number of neighbors used in the analysis (Fig. 4b, d). Reflecting chromatin compaction, the average distance to neighbors increases after 1 hour of OND, which resolves upon restoration of normoxia and the energy source (Fig. 4c). These effects become more apparent when further adjacent neighbors are included in the analysis, at least up to 500 nearest neighbors (Fig. 4d).

### OND decreases the susceptibility of chromatin to DNAseI digestion

**Fig. 5:**
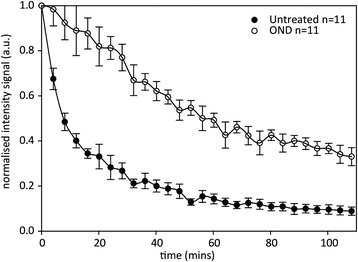
OND induces chromatin compaction as determined by resistance to digestion by DNAseI. HL-1 cells, either untreated or subject to 1 hour of OND, were fixed, permeabilized and stained with 5 μM DRAQ5 for 30 minutes. Cells were then digested with 5 U/ml DNAseI at 37 °C with cellular fluorescence measured on a confocal microscope, with images generated every 4 minutes, observing 11 cells in total for each experimental condition, *a.u.* arbitrary units

We next utilized a biochemical approach to confirm that OND treatment for 1 hour does indeed provoke compaction of chromatin. We estimated the access of a large molecular weight probe, DNAseI (30 kDa), to chromatin. DNA in fixed and permeabilized untreated or OND-treated cells was preloaded for 30 minutes with DRAQ5, a selective DNA interchelating dye [47], and then subjected to digestion with DNAseI, with cellular fluorescence continuously measured on a confocal platform. Digestion of DNA provokes liberation of DRAQ5, with the rate of decrease in DRAQ5 fluorescence dependent upon on the extent of chromatin compaction. As shown in Fig. 5, untreated cells show a triphasic response to DNAseI treatment, with a highly accessible subfraction of chromatin, approximately 50 % of the total, predominating the kinetics of the first 15 minutes of the time-course. A more compact, but nevertheless digestible, fraction then defines the following 40 minutes of digestion, with a residual proportion of chromatin, some 10 % of the total, predominantly resistant to DNAseI digestion. In contrast, OND cells show a biphasic response, with a compact but digestible fraction dominant for the first 60 minutes of digestion, followed by a fraction of chromatin (around 30 % of the total) that is relatively resistant to DNAseI digestion. OND cells do not exhibit a rapidly digested fraction of chromatin, as observed in untreated cells. These results confirm and extend our SMLM observations that OND induces profound compaction of chromatin, particularly of loosely condensed chromatin.

### OND reduces cellular ATP levels, inhibits transcription, redistributes polyamines to the nucleus and restricts access to histones

**Fig. 6:**
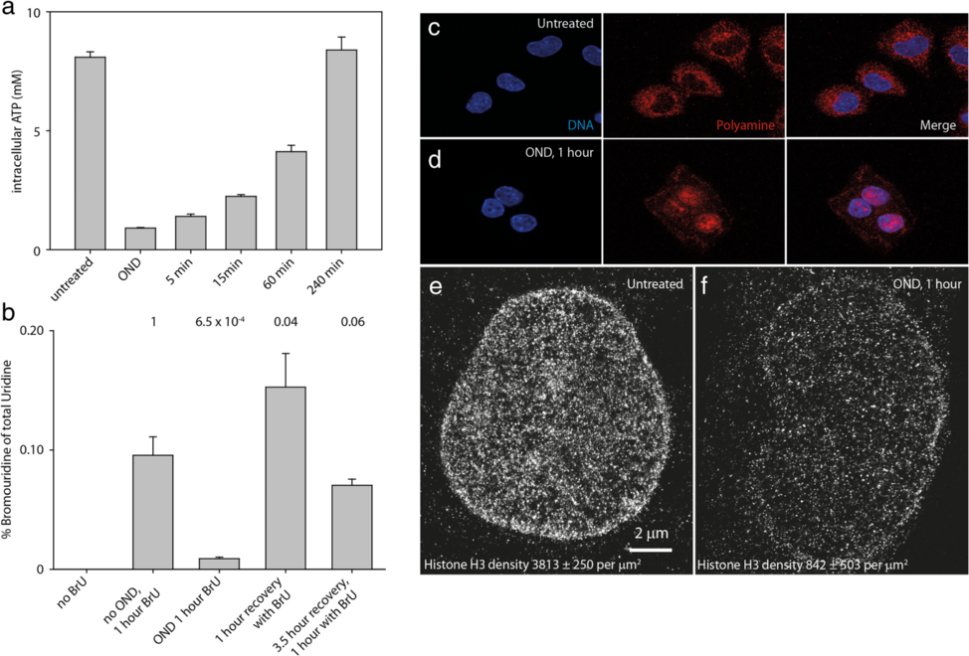
OND depletes intracellular ATP levels, inhibits transcription, induces relocation of the cellular polyamine pool to the nucleus and reduces the staining density of histone H3 with antibody. **a** The intracellular concentration of ATP in untreated, OND-exposed and recovering cells was determined using a luciferase dependent assay. **b** Global rates of transcription, determined by the incorporation of bromouridine into RNA, in untreated cells, cells under OND and cells recovering from OND are presented. HL-1 cells, either untreated or subjected to 1 hour of OND, were fixed, permeabilized and stained either with anti-polyamine antibody (**c**, **d**) or with anti-total H3 antibody (**e**, **f**) and counterstained with the fluorescent DNA binding dye Hoechst 33342. Cells were then examined using confocal microscopy. The content of immunostained histone H3 was evaluated by SMLM in untreated (**e**) and OND-treated (**f**) HL-1 cells. *BrU* bromouridine. The error bars represent the standard deviation of three independent samples

We then postulated that chromatin compaction induced by OND is consequent upon ATP depletion. Under normal conditions, divalent cations and polyamines associate with the polyphosphate group of ATP. However, if ATP levels are reduced, these may, by mass-action, relocate to the sugar-phosphate backbone of nucleic acid, thereby promoting chromatin compaction through effecting charge shielding. OND reduces intracellular ATP levels by 90 %, which recovers upon cessation of OND with kinetics similar to that of chromatin relaxation (Fig. 6a). Furthermore, OND promotes a global decrease of transcription by approximately 90 %, as estimated by mass spectrometric determination of bromouridine incorporation into nascent RNA (Fig. 6b). We then described the distribution of the intracellular polyamine pool using immunocytochemistry. Anti-polyamine staining of untreated HL-1 cells results in a punctate, predominantly cytoplasmic distribution with a low level of intranuclear staining (Fig. 6c). This most likely reflects ATP-rich mitochondria present in the cytoplasm of cardiomyocytes. In contrast, OND treatment for 1 hour results in the transfer of a significant part of the cellular polyamine pool to the nucleus (Fig. 6d) with particularly intense staining of RNA rich nucleoli. Additionally, SMLM of histone H3 indicates that, in comparison with untreated cells (Fig. 6e), OND treatment (Fig. 6f) reduces the apparent density of chromatin-associated histone H3 in the nucleus from 3813 ± 250 per μm^2^ to 842 ± 503 per μm^2^, whereas levels observed in the cytoplasm remain similar at 250 per μm^2^. Furthermore, the localization density achieved for total H3 is much lower than for DNA binding dyes, and is insufficient to discern OND-induced chromatin compaction.

### FRAP indicates that core histones are not displaced from chromatin upon OND-induced compaction, and that OND decreases the mobility of the linker histone H1

**Fig. 7:**
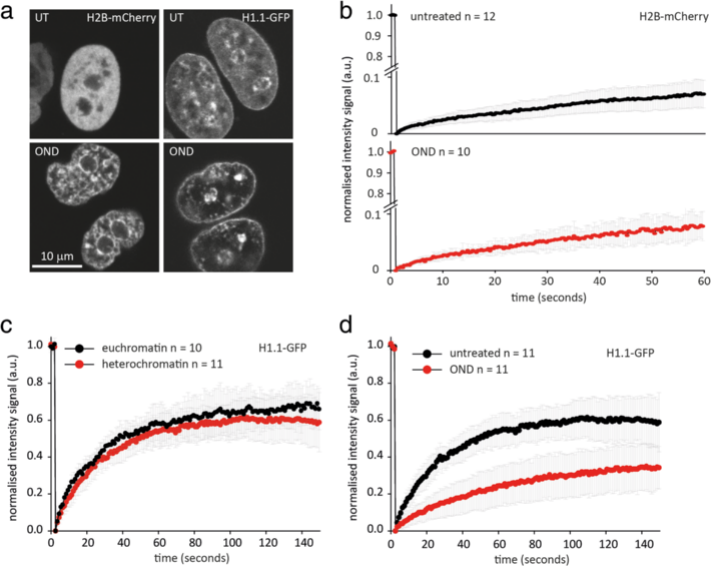
OND does not induce core histone displacement from chromatin but does decrease the mobility of the linker histone H1. We first demonstrated that HeLa cells stably transfected with either histone H2B-mCherry or histone H1.1-green fluorescent protein (GFP) respond to 1 hour of OND by undergoing chromatin compaction. **a** Comparison of untreated (*UT*) cells (*top panels*) with cells exposed to 1 hour of OND (*bottom panels*) by confocal microscopy clearly indicates that chromatin of HeLa cells compacts upon OND treatment. **b** We then evaluated the mobility of the core histone H2B using FRAP on untreated (*upper panel*) and on OND-treated (*lower panel*) cells. The recovery after photobleaching was extremely slow for both conditions, indicating that OND does not induce displacement of H2B from chromatin. **c** We then evaluated the mobility of the linker histone H1 in untreated and OND-treated HeLa cells. As previously reported [49, 50], histone H1 is mobile, and is somewhat less mobile in heterochromatin than in euchromatin. **d** OND-induced chromatin compaction dramatically reduces the mobility of histone H1, indicating that the extent of chromatin compaction in OND is considerably higher than that between euchromatin and heterochromatin

We wished to discriminate between possible explanations underlying the approximately 80 % reduction in histone H3 staining upon OND treatment, as determined by SMLM. Potentially, this observation could arise through compaction restricting the accessibility of antibody to chromatin and/or through the direct loss of core histones from chromatin. By inference, histone loss from chromatin would liberate a highly mobile pool of histones, in contrast to their limited mobility when present in chromatin. We therefore used FRAP on live cells to estimate the mobility of histone H2B labeled with mCherry in untreated HeLa cells and HeLa cells in an ischemic environment. We selected H2B, which along with H2A, and in contrast to H3 and H4, exhibits significant exchange [48]. Consequently, FRAP analysis of H2B-mCherry is an appropriate marker for estimating OND-induced displacement of core histones. As shown in Fig. 7a, HeLa cells undergo chromatin compaction when subjected to 1 hour of OND, and significantly, H2B-mCherry retains a structured nuclear distribution, suggestive of chromatin compaction, indicating that a widespread release of core nucleosomes from chromatin does not occur upon OND treatment. FRAP measurements (Fig. 7b) of the mobility of H2B-mCherry confirm that OND does not increase the mobility of this core histone.

We next assessed the mobility of the linker histone H1.1 in HeLa cells, which maintains higher order chromatin structure through binding to extranucleosomal DNA. H1 exchanges continuously, with a residence time of a few minutes, even within heterochromatin [49, 50]. We firstly confirmed these observations in untreated HeLa cells, demonstrating that the mobility of histone H1.1-green fluorescent protein (GFP) is higher in euchromatin compared with heterochromatin (Fig. 7c). In agreement with the extensive compaction of chromatin induced by OND treatment for 1 hour, the mobility of histone H1.1-GFP is significantly reduced upon treatment (Fig. 7d), demonstrating that (a) displacement of histone H1.1 does not occur and that (b) OND induces chromatin compaction to an extent that restricts the exchange of histone H1.1, and that the extent of this compaction exceeds the extent of the difference between euchromatin and heterochromatin. In conclusion, OND does not induce displacement of core histones but does reduce the mobility of the linker histone H1.1. This suggests that the decrease in density of immunostained H3 in OND results from compaction of a substantial fraction of chromatin to an extent that excludes penetrance by antibodies.

### OND-induced chromatin compaction can be estimated by cytometry, provokes histone deacetylation and decreases the internal structure of cells

**Fig. 8:**
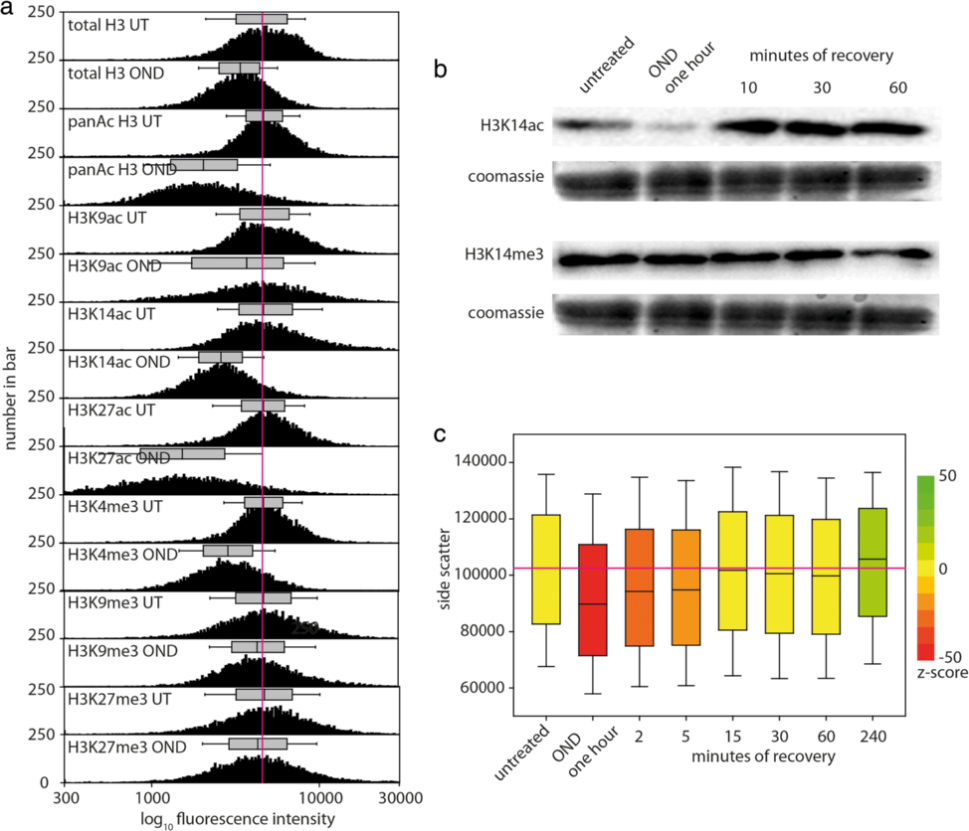
OND reduces access to chromatin by anti-histone antibodies and induces deacetylation of histones and a reduction in cellular granularity. HL-1 cells, either untreated or subjected to 1 hour of OND, were trypsinized to produce a monodisperse suspension, fixed, permeabilized, washed and immunostained with anti-histone H3 antibodies, as indicated. Cytometric analysis was performed on a minimum of 10^4^ cells. **a** A comparison of the staining intensity of untreated (*UT*) and OND cells; each data pair is normalized to the median of the untreated total H3 with the median and range of the distribution shown above each histogram. **b** Western blot analysis of total H3K14ac and of H3K14me3 across the experimental time-course. **c** The distribution of side-scatter measurements, which are proportional to internal cellular granularity, is presented for 10^4^ cells as box plots showing the median, 25 % and 75 % intervals as boxes and the 5 % and 95 % intervals as whiskers. The median value for untreated cells is shown as a *horizontal line* through all box plots. The z score between the untreated cell population and each other experimental condition, determined by the Mann-Whitney ranked sum test, is indicated by the color of the box according to the key on the right

We further explored OND-induced chromatin compaction using cytometric analysis of histone H3, and of post-translationally modified histone H3 variants. We reasoned that antibodies would stain compacted chromatin to a lesser extent than chromatin in untreated cells, thereby facilitating a semi-quantitative evaluation of the extent of OND-induced histone chromatin compaction. Furthermore, ischemia results in a general decrease of histone H3 [51–55] and H4 [56–58] acetylation levels. We therefore anticipated that OND should provoke a general reduction in antibody staining against histone marks, due to compaction restricting antibody access and, moreover, that this effect should be especially pronounced for acetylated histone marks. In accordance with these considerations, OND induces a considerable reduction in staining of total histone H3, pan-acetylated H3, H3K9ac, H3K14ac, H3K27ac, H3K4me3, and, to a lesser extent, of H3K9me3 and H3K27me3 (Fig. 8a). Accessible histone marks, such as acetylated H3 variants or trimethylated H3K4, are affected by OND to a greater extent compared with either total H3 or, in particular, with histones present in compacted chromatin, such as H3K9me3 and H3K27me3. The kinetics of histone acetylation and methylation of lysine 14 of histone H3 is shown in Fig. 8b. One hour of OND induces a dramatic loss of H3K14 acetylation, which rapidly recovers upon restitution of oxygen and nutrients. H3K14 trimethylation exhibits little change through the experimental time-course. Similar results are obtained by confocal evaluation of cells stained with anti-H3K9ac (Figure S9 in Additional file 2) and with anti-H3K14ac (Figure S10 in Additional file 2); OND induces a profound loss of histone acetylation that recovers several minutes after release from OND. A useful feature of cytometric analysis is the detection of side-scattered blue light, which is proportional to the granularity or internal complexity of the cell. Side-scatter (SSC) is a measurement of mostly refracted and reflected light that occurs at any interface within the cell where there is a change in refractive index [59]. We anticipated that OND-induced compaction of chromatin should result in a change in detected SSC, providing an independent methodology reporting the effect of OND on chromatin. Importantly in the context of this analysis, OND does not induce a significant change in nuclear volume (Results in Additional file 2). As shown in Fig. 8c, OND induces a reduction in SSC that recovers on restoration of normoxia and nutrients. In keeping with our previous observations, SSC measurements are significantly higher 4 hours post-recovery compared with untreated cells.

## Discussion

Ischemia is a defining event in the prevalent causes of morbidity in humans, including stroke, myocardial infarction and cancer. We show, using single molecule localization of DNA binding dyes, that the nuclear architecture of immortal cardiomyocytes undergoes dramatic and reversible compaction under experimental conditions mimicking transient ischemia followed by reperfusion. While functionally reversible compaction changes under ATP depletion conditions have previously been observed [32, 60, 61] using conventional microscopy approaches, this report quantitatively describes the nanoscale condensation of chromatin in myocardiac cells at single molecule resolution and has sufficient spatial resolution to qualitatively describe the extent of chromatin compaction. These analyses comprehensively reveal the extent, mechanism and reversibility of OND-induced chromatin compaction and have been confirmed by alternative analytical procedures.

The extent of compaction indicates that chromatin undergoes a phase transition under OND; that is, chromatin changes from a structurally more open, ‘disordered’ state to a structurally more closed, ‘ordered’ state. This is in keeping with the partial exclusion of DNA binding dye from chromatin upon OND and is consistent with the random obstacle network model recently proposed by Baum et al. [62]. OND induces a previously undescribed subnuclear configuration composed of discrete, DNA dense, atoll-like structures interspersed between large chromatin-sparse voids. Moreover, OND provokes an extensive depletion of ATP and a relocation of the intracellular polyamine pool from the cytoplasm to the nucleus. Mechanistically, chromatin compaction is consistent with OND-induced liberation of polyamine and, correspondingly, of divalent cations. This process directly links cellular energy status with chromatin architecture. Upon cessation of ischemic-like conditions, the nuclear structure of cardiomyocytes undergoes relaxation, within a period of tens of minutes. Moreover, chromatin adopts a more open configuration, compared with untreated cells, several hours after release from OND. This effect may be of consequence in influencing the epigenetic reprogramming of cells.

In the presence of multivalent cations, high molecular weight DNA undergoes a dramatic condensation to a compact, usually highly ordered toroidal structure, with experimental evidence showing that DNA condensation occurs when about 90 % of its charge is neutralized by counterions [63]. ATP exists in the cell predominantly as a complex with Mg^2+^. Consequently, OND-mediated reduction in intracellular ATP concentrations increases intracellular availability of Mg^2+^ [64] and may promote chromatin compaction through divalent cation-mediated charge shielding of phosphate groups in DNA. Experimentally increasing the osmolarity of culture medium [30] or increasing the exposure of detergent-permeabilized cells to divalent cations, but not monovalent cations, provokes chromatin compaction as evaluated by either confocal microscopy or FLIM-FRET [32]. Similarly, as the ATP–Mg^2+^ complex sequesters intracellular polyamines, principally spermine and spermidine [65], a reduction in ATP levels results in the intracellular polyamine pool transferring to chromatin by mass action, thereby further enhancing condensation [32]. In keeping with these proposed effects, transient ATP depletion by inhibition of oxidative phosphorylation with azide in SW13 and HeLa cells induces an increase in the volume of the interchromosomal compartment, as observed by confocal microscopy [60].

Chromatin primarily consists of DNA wrapped around the core histone complex [66]. Additionally, OND results in a profound loss of active histone marks, particularly acetylation and H3K4 trimethylation. This raises the issue of how the histone code, particularly for active genes, is reinstated during recovery from an ischemic environment and may provide new insight into the phenomenon of ischemic preconditioning, where pretreatment of an organ with short periods of ischemia has a protective effect on subsequent ischemic insult [67]. The degree of chromatin relaxation upon recovery from OND exceeds that of untreated cells, indicating that chromatin may adopt a more transcriptionally permissive configuration compared with either untreated or OND-treated cells. This could arise as a consequence of intracellular ATP levels exceeding the divalent and polycation pool of the cell upon recovery from OND, such that chelation of the intracellular polyamine and divalent cation pool by ATP exceeds the amount present under continuous normoxic and nutrient-rich conditions.

There is an intimate connection between chromatin architecture and the functional output of chromatin. Advanced technologies are revolutionizing understanding of chromosome organization, and are progressing understanding of the influence of spatial organization on transcription, replication and repair [68]. Ischemic conditions provoke an extreme level of chromatin compaction, as witnessed, for example, by the extensive development of chromatin-free areas within the nucleus and in the restriction of linker histone H1 activity mobility that far exceeds that within heterochromatin. However, the methodologies we describe, if utilized in conjunction with specific labeled genomic regions, could be developed to probe local DNA configurations. The defining characteristic of the SMLM we have performed is the labeling density that can be achieved using DNA binding dyes. This allows resolution of chromatin nanostructure at a scale appropriate to inform on regulatory events.

The extent and reversibility of chromatin compaction induced by OND suggests that the impact of ischemia could be constrained by targeting biochemical events that are required for chromatin condensation. In this light, pan-inhibition of histone deacetylase (HDAC) activity is efficacious in animal models of cerebral ischemia [69] and specific knockdown of HDAC3 or HDAC6 promotes survival of cortical neurons in an in vitro model of ischemia employing oxygen and glucose deprivation [70]. Increased HDAC activity was reported in a mouse model of cardiac ischemia and inhibition of HDACs by trichostatin A treatment significantly reduced the infarct size [71]. Moreover, OND-induced compaction of chromatin may explain the observed increase of histones in serum that occurs in catastrophic ischemic events. Alternative strategies could be to chelate intracellular divalent cations or to limit the production of polyamines — for example, through inhibition of ornithine decarboxylase activity. Intriguingly, extensive pre-clinical evidence indicates that this strategy is of benefit in restricting the growth of solid tumors [72] and inhibiting ornithine decarboxylase protects *Drosophila* against hypoxia-induced reduction in lifespan [73]. In summary, ischemic conditions induce a rapid compaction of chromatin, which is associated with a general inhibition of transcription [6]. Correspondingly, the nuclear architecture senses and responds to environmental conditions through structural rearrangements. Defining and understanding these effects offers a diverse range of tractable targets for therapeutic intervention in human disease.

## Conclusions

Experimental ischemia induces a profound compaction of chromatin, which is reversible upon restoration of normoxia and nutrients. Ischemic conditions lower intracellular ATP levels, result in the redistribution of the intracellular polyamine pool into the nucleus and induce a large reduction in the rate of synthesis of RNA. Upon recovery from ischemia induced chromatin compaction, chromatin transiently acquired a more open and transcriptionally active configuration as compared to untreated cells.

## Materials and methods

### Cells and cell culture

HL-1 cells are an immortal mouse cardiomyocyte cell line derived from a murine atrial tumor which retain the morphology and gene expression profile of adult cardiomyocytes and the ability to contract [44]. They were cultured in gelatin/fibronectin-coated dishes in Claycomb medium (Sigma) supplemented with 2 mM glutamine (Gibco), 0.1 mM norepinephrine (Sigma-Aldrich), 10 % fetal bovine serum (Sigma-Aldrich) in 5 % CO_2_, 37 °C and 95 % humidity. Cells were passaged every 3 days as described [44]. For microscopic analysis, HL-1 cells were grown on coated glass cover slips (Assistant, 20 × 20 mm) in six-well plates to a density of 50 %. All other experiments were performed on confluent cells.

### Oxygen nutrient deprivation

HL-1 cells were washed twice with phosphate-buffered saline (PBS; Gibco) and placed in a hypoxia chamber (Whitley Hypoxystation H35) with 1 % O_2_, 5 % CO_2_, 94 % N_2_ at 37 °C and 70–85 % humidity. An ischemic environment was simulated by incubating cells in 115 mM NaCl, 12 mM KCl, 1.2 mM MgCl_2_, 2 mM CaCl_2_, 25 mM HEPES and 5 mM deoxyglucose; this solution was pre-equilibrated to 1 % O_2_ prior to use. Cells were incubated for 1 hour under these conditions, after which they were washed with PBS then returned to Claycomb media under normoxic conditions. Experimental evaluation was usually performed on untreated cells, cells subjected to 1 hour of OND and on recovering cells at 5, 15, 60 and 240 minutes following OND. Recovery was designed to mimic reperfusion after an ischemic event. Non-recovered OND-treated cells were harvested and fixed in a hypoxic atmosphere. All buffers used for the preparation of such samples were equilibrated to 1 % O_2_ in advance. Untreated cells were kept under normal culture conditions until fixation.



Schematic representation of the time-course employed to evaluate the effect of OND, and subsequent recovery, on HL-1 cells.

### Immunofluorescence analysis of histones, Lamin B1 and DNA by confocal microscopy

Cells grown on coated cover slips in six-well plates were treated with 2 ml of OND buffer or with Claycomb medium as indicated. Cells were then washed twice with PBS, fixed in 1 ml ice cold methanol for 10 min, washed with PBS and permeabilized with 1 ml PBS containing 0.3 % Triton X-100 (Sigma-Aldrich) and 0.3 % Tween-20 (Sigma-Aldrich) for 10 minutes at room temperature. Blocking was performed with 1 ml blocking buffer (5 % bovine serum albumin (BSA) and 0.1 % Tween-20 in PBS) for 1 hour at room temperature. For the antibody labeling, cells were incubated with anti-H3 (Abcam, 1 μg/ml), anti-H3K14ac (Cell Signaling, 1:500), anti-Lamin B1 (Abcam, 1 μg/ml) or anti-polyamines (Abcam, 10 μg/ml) overnight at 4 °C in 500 μl blocking buffer. After incubation with primary antibody, cells were washed three times with 1 ml wash buffer (PBS containing 0.1 % Tween-20) and incubated with AlexaFluor 488 conjugated secondary antibody (Invitrogen, 2 μg/ml) for 1 hour in 1 ml wash buffer, followed by three washes with wash buffer. DNA was stained with Hoechst 33342 (0.5 μg/ml) for 20 minutes at room temperature and washed three times with 1 ml PBS. Cells were then embedded in 10 μl of glycerol. For analysis, a Leica SP5 II confocal system (Leica Microsystems GmbH) with a 63× oil immersion NA1.4 objective lens was used, and 1024 × 1024 images were acquired using a pinhole size of 1.0 Airy units, 60–100 nm pixel pitch.

### Fluorescence recovery after photobleaching

FRAP experiments utilized HeLa cells stably transfected with mCherry-H2B or with GFP-H1.1. Live cell experiments were performed either in OND buffer or in RPMI 1640 without phenol red (Life Technologies) containing 10 % fetal bovine serum (Gibco). OND samples were tightly sealed using Picodent Twinsil two component glue (Wipperfuerth, Germany) within a hypoxia chamber prior to FRAP analysis. The parameters used for the acquisition of FRAP data are listed in Table 1.

**Table 1:** Parameters used for the acquisition of FRAP data

	H2B-mCherry	H1.1-GFP
FRAP image acquisition	256 × 256 pixels	256 × 256 pixels
	Effective pixel size 96.5 nm	Effective pixel size 96.5 nm
	Excitation: 6 % power of 561 nm	Excitation: 13 % power of 488 nm
	Emission: 602–711 nm	Emission: 500–591 nm
Bleaching protocol	One 256 × 256 pixel scan in 2 × 2 μm, 80 % 561 nm laser	100 ms point bleach, 80 % 488 nm laser
Time/frame	0.328 s	1 s
FRAP read-out	2 × 2 μm square ROI	1.5 μm in diameter circle ROI

Data were processed as described in Trembecka-Lucas et al. [74] with some modifications. Eleven individual FRAP measurements were performed for each data set. Each FRAP acquisition was aligned using the StackReg ImageJ plugin to compensate for movement of the bleached area during fluorescence recovery [75]. Fluorescence recovery after photobleaching was then analyzed within a respective ROI using ImageJ and corrected for bleaching throughout the experiment by acquisition of bleaching curves for either H1.1-GFP or H2B-mCherry in independent measurements in both buffers to accommodate possible differences in bleaching rates.

### Flow cytometry analysis of histone marks

Cells were grown to confluency in 10-cm diameter culture dishes, then subjected to the ischemia/reperfusion protocol as previously described, using 10 ml of OND solution or Claycomb medium. Cells were then washed twice with 10 ml PBS, trypsinized with 1 ml 0.25 % Trypsin (Gibco) and vigorously resuspended in 5 ml of PBS containing soybean trypsin inhibitor. The cells were centrifuged at 250 × g for 5 minutes and washed once in 10 ml PBS and the cell pellet was resuspended in 1 ml ice cold methanol for 10 minutes to fix the cells. Cells were again centrifuged and then permeabilized for 10 minutes in PBS containing 0.3 % Triton X-100 and 0.3 % Tween-20. After a further centrifugation, the cell pellet was resuspended in 500 μl PBS and the cell density was estimated using an automated cell counter (BioRad). One million cells were resuspended in 300 μl fluorescence-activated cell sorting (FACS) buffer (PBS with 0.1 % Tween-20, 1 % BSA) containing anti-H3 (Abcam, 3 μg/ml), anti-H3K14ac (Cell Signaling 1:300), anti H3K9ac (Cell Signaling, 1:300), anti-H3K27ac (Abcam, 3 μg/ml), anti-pan-acetylated H3 (Merck Millipore, 3 μg/ml), anti-H3K4me3 (Merck Millipore, 3 μg/ml) and anti-H3K27me3 (Active Motif, 3 μg/ml) and incubated for 1 hour. Cells were washed three times with 1 ml FACS buffer and incubated with 1 μg/ml AlexaFluor 488 conjugated secondary antibody (Invitrogen) for 45 minutes. After a further three washes with FACS buffer, cells were resuspended in 300 μl PBS and the fluorescence intensity of the cell population was analyzed using a BD LSRFortessa cytometer (BD Biosciences). Cells were gated so that only events from discrete single cells were counted, with 10^4^ events recorded per experimental condition.

### ATP determination

Cells were grown to confluency in 3.5-cm diameter culture dishes and OND applied to selected samples as described previously, using 2 ml of ischemic salt solution as required. After OND, cells were washed twice with PBS, and ATP extracted with boiling water as described by Yang et al. [76]. ATP was determined using a bioluminescence assay utilizing recombinant firefly luciferase and its substrate D-luciferin (ATP Determination Kit, Invitrogen).

### Bromouridine labeling of nascent RNA

Newly synthesized RNA was pulse labeled by incubating confluent 10-cm culture dishes with 2 mM bromouridine (BrU; Sigma Aldrich) for 1 hour, either under normal culture conditions, under OND, or after 1 hour of recovery from OND. To estimate background incorporations, cells were analyzed without exposure to BrU. All experimental conditions were performed in triplicate. Following BrU pulse labeling, RNA was extracted using Trizol (Ambion) following the manufacturer’s instructions and following digestion to nucleosides with nuclease P1 (Roche), U snake venom phosphodiesterase (Worthington) and alkaline phosphatase (Fermentas) as described by Kellner et al. [77]. RNA nucleosides were subjected to liquid chromatography-mass spectrometry analysis. Separation was performed on an Agilent 1290 UHPLC system equipped with a ReproSil 100 C18 column (3 μm , 4.6 × 150 mm, Jasco GmbH) maintained at 30 °C. Identification and quantification of nucleosides was performed on an Agilent 6490 triple quadruple mass spectrometer.

### Determination of nuclear volume

Cells were cultured in six-well plates on coverslips and subjected to 1 hour of OND as described. Cells were then fixed for 10 minutes on ice with methanol, permeabilized for 10 minutes in PBS containing 0.3 % Triton and stained with Hoechst 33342 (2 μg/ml, Sigma). Samples were embedded in glycerol and the nuclear volume was calculated by reconstruction of nuclear z-stacks following acquisition on a Leica SP5 II confocal system (Leica Microsystems GmbH) using a step size of 0.21 μm. A 1.4 NA 63× oil objective was used. The Imaris software package (Bitplane) was used to calculate nuclear volume using the following parameters: surface grain size, 0.170 μm; threshold absolute intensity, 14.6702; distance to image border xy, 0.429 μm; volume, above 200 μm. Statistical analysis was performed using the Mann-Whitney rank sum test.

### Western blotting

Histones were extracted from 10-cm dishes of confluent HL-1 cells after OND and OND plus 10, 30, or 60 minutes of recovery. The cells were washed with PBS supplemented with 5 mM sodium butyrate to prevent deacetylation. The cells were collected in 800 μl of Triton extraction buffer containing PBS with 0.5 % Triton X-100, 2 mM phenylmethylsulfonyl fluoride (PMSF) supplemented with 5 mM sodium butyrate and protease inhibitor. Cytoplasmic lysis was performed for 10 minutes on ice followed by a 10-minutes centrifugation step at 2000 rpm at 4 °C. The resulting nuclei pellet was resuspended in 100 μl of 0.2 N hydrochloric acid and histone extraction was performed overnight at 4 °C with rotation. After a 2000 rpm centrifugation step, the supernatant was collected and protein concentration was determined by Bradford assay. Five micrograms of histones were diluted in laemmli loading buffer and boiled for 5 min. The samples were run on a 12.5 % SDS gel and subsequently blotted on nitrocellulose membranes for 1 hour at 100 V at 4 °C. Ponceau staining was used as a loading and transfer control. The membranes were blocked in 5 % BSA in TBST buffer for 1 hour, and incubation with primary antibody (H3K14ac, Abcam, 1:5000; H3K14me3, Signalway Antibody, 1:5000) was done overnight at 4 °C. After three TBST washes the secondary antibody conjugated to horse radish peroxidase was incubated for 45 minutes and washed off three times. The blots were developed with 1 ml ECL reagent (Invitrogen) per blot and pictures were taken under a ChemiDoc (Biorad).

### DNAseI digestion of chromatin

Time-course in situ digestion assays to determine the relative resistance of chromatin in untreated and OND-treated cells to DNAseI were performed using a Leica SP5 confocal microscope (Leica, Wetzlar, Germany) at 37 °C. HL-1 cells were seeded on an IBIDI eight-well chamber, subjected to 1 hour of OND, or not, then fixed for 15 minutes with 4 % paraformaldehyde, followed by permeabilization using 0.3 % Triton X-100 in PBS. Cells were stained with 320 μl of 5 μM DRAQ5 (Life Technologies) for 30 minutes then subsequently washed twice with PBS. PBS was replaced with 150 μl 1× DNase buffer (NEB) and placed on to the microscope stage. DNaseI (150 μl of 10 U/ml) in DNase buffer was then diluted to 5 U/ml final concentration and time-lapse measurement of DRAQ5 fluorescence was initiated. Images were taken every 4 minutes with autofocus stabilization, and were acquired with the following settings: 7 % 633 nm laser excitation, 643–749 nm emission range, 512 × 512 resolution (voxel size 246 × 246 × 481.5 nm), 2 AU pinhole, 600 Hz scanner speed.

### Localization microscopy

#### Sample preparation for SMLM

HL-1 cells on coverslips were cultured, fixed and permeablilized as described. Samples were washed twice with PBS then incubated for 40 minutes with 1 μM Vybrant DyeCycle Violet (Life technologies), followed by a further two washings with PBS. For SMLM imaging of YOYO-1, cells on coverslips were permeabilized, incubated with 0.5 U/ml RNase A and 20 U/ml RNase T1 (Ambion, USA) for 1 h at 37 °C, then stained with 0.02 nM YOYO-1 in 1 ml PBS for 30 min. Cells were then washed twice with PBS and embedded in 20 μl PBS containing 40 μg/ml glucose oxidase, 0.5 mg/ml catalase and 10 % (w/v) glucose. SMLM was performed after 300 minutes once the majority of dye dissociates from the DNA to the interior of the nucleus (Figure S4 in Additional file 2). For SMLM imaging of AlexaFluor 647-stained samples, an imaging buffer of 40 μg/ml glucose oxidase, 0.5 mg/ml catalase, 10 % (w/v) glucose and 50 mM (for Lamin B1 staining) or 100 mM (for histone imaging) mercaptoethylamine in 60 % (v/v) glycerol and 40 % (v/v) PBS was used. For SMLM imaging of Vybrant DyeCycle Violet an imaging buffer consisting of 40 μg/ml glucose oxidase, 0.5 mg/ml catalase, 10 % (w/v) glucose in 80 % glycerol and 20 % PBS was used. For two-color imaging of DNA and AlexaFluor 647, the imaging buffer was further enriched with 3 mM mercaptoethylamine (a concentration that facilitates blinking of the cyanine dye AlexaFluor 647 without affecting blinking of Vybrant DyeCycle Violet [43]. The appropriate buffer (20 μl) was placed on a glass slide after which the coverslip with fixed cells was placed upside-down on the droplet. The coverslip was bonded to the slide with biologically inert, impervious dental paste (Picodent Twinsil) prior to SMLM imaging.

#### SMLM measurements

The SMLM configuration has been described previously [36]. Briefly, the custom-built microscope was equipped with a single objective lens (Leica Microsystems, 1.4 NA, 63× oil immersion with a refractive index of 1.518) and a 12 bit, air-cooled CCD camera (PCO, Sensicam QE, effective pixel size in the sample region corresponds to 102 nm). For fluorescence discrimination, emission filters used for Vybrant DyeCycle Violet, YOYO-1 and AlexaFluor 488 were bandpass filter 525/50 nm (Chroma) and for AlexaFluor 647 long pass 655 nm (Chroma). Widefield acquisitions were performed with homogeneous illumination of the whole field of view, as achieved by 6.7-fold expansion of the laser beam. SMLM images were acquired upon illumination with a collimated laser beam covering an area of ~25 μm diameter in the imaging plane (full width at half maximum of Gaussian profile). For single color imaging of AlexaFluor 647, a 647 nm laser (LuxX diode laser, Omicron, Germany) was used at 60 mW (measured in a sample plane), and 25,000 frames with an exposure time of 25 ms were acquired for each SMLM experiment. Stained DNA (Vybrant DyeCycle Violet, YOYO-1) in HL-1 cells was excited using a 491 nm laser (Calypso 05 series, Cobolt, Sweden). For Vybrant DyeCycle Violet 30,000 frames with 50 ms exposure time were acquired at 70 mW laser power (sample plane), and for YOYO-1 30,000 frames with an exposure time of 50 ms were acquired at 30 mW laser power (sample plane). In the dual color experiments, imaging of AlexaFluor 647 (9000 frames with 25 ms exposure time, 647 nm excitation, 60 mW in the sample plane) was performed prior to imaging of DNA stained with Vybrant DyeCycle Violet. For imaging of AlexaFluor 488 23,000 frames with camera exposure time of 25 ms were acquired at 70 mW 491 excitation.

#### Data analysis and post-processing

SMLM data analysis was performed using fastSPDM, a custom software package written in Matlab [78] to extract single molecule fluorophore positions from raw tiff data stacks. First, an initial background image was calculated by averaging over eight frames. Noise in the background was calculated assuming a Poisson noise model, i.e., the standard deviation of the noise is given by STD = (background)^1/2^. Looping through each of the acquired frames, the background was subtracted from the raw data, yielding a difference image. Initial estimations of the positions of single fluorophore signals were detected with pixel accuracy in the difference images (after smoothing with a 3 × 3 mean filter) based on a threshold factor (TF) defined as follows: Only signals with a peak intensity I0 higher or equal to (TF – 1) × STD were considered. Subpixel accuracy positions were extracted from a 7 × 7 ROI around the initial estimation of each signal by calculating the center of intensity. Overlapping signals detected as radially increasing intensity as we move away from the center pixel of the ROI were clipped, yielding a refinement of the positions. If clipping results in more than 30 % loss of accumulated signal within the ROI, then the signal is discarded. From the remaining signals, a list of localizations was generated containing information on x, y-position, photon count, and localization precision σ_loc_ [79] for each signal, where localization precision of a point emitter is defined as follows:

$$ {\sigma_{\mathrm{loc}}}^2=\frac{s^2+{p}^2/12}{N}+\frac{8\pi {s}^4{b}^2}{p^2{N}^2} $$

where *N* corresponds to number of photons, *s* is a standard deviation of Gaussian point-spread function, *p* is pixel size, and *b* is background noise. The difference image was further used to adjust the background image for the next frame as follows: Values larger than STD were clipped from the diff image, and the result was scaled by a factor of 1/8 and added to the previous background image. This new estimate of the background image was made positive by clipping values less than 0.

For analysis of the SMLM data, TF values of 3 (DNA stained with Vybrant DyeCycle Violet or YOYO-1) or 3.5 (AlexaFluor 647) were used. The initial list of localizations as described above was altered by joining single molecule signals occurring in consecutive frames (search radius = 2.5 < σ_loc_>). No further filtering of the list of localizations (e.g., based on localization precision) was performed. Such filtering of the list of localized signals in PALM/STORM-related approaches based on the width of the detected signal is often necessary because even weak signals are easily picked up if the background intensity is close to zero. Assuming a Poisson noise model for the detected photons, then the noise in the detected background is also close to zero. In our approach, single molecule signals of the DNA dyes are detected on top of a relatively high background (average of 150 counts, i.e., 300 photons). In this case, the x-y broadening of the detected fluorescence signals as we move away from the focal plane is sufficient to reject these out-of-focus signals, as they become hidden in the noise level.

Prior to final reconstruction, the drift in the images occurring during acquisition was determined from the list of localizations using correlation of reconstructions of up to 100 subsets. The drift determined in this way amounts to approximately 150 nm/h, and was used to correct the list of localizations. The shift between drift-corrected subsets of data usually did not exceed a standard deviation of σ_*drift*_ = 10 nm.

For visualization/reconstruction of the SMLM data, Gaussian blurring was used: All single fluorophore positions were blurred with their respective localization precision σ_loc_ (AlexaFluor 647) or with the average localization precision < σ_loc_ > (DNA/SMLM data) to generate a SMLM reconstruction. All reconstructed images were generated with a pixel size corresponding to 5 nm.

The single molecule photon count of DNA dyes has an average of about 1500 photons, i.e., much less than the brightest dSTORM/SMLM fluorophores. The resolution σ_*total*_ in the DNA SMLM data was estimated from the photon count-dependent localization precision using the formula:

$$ {\sigma}_{total}=\sqrt{{\left({\sigma}_{loc}\right)}^2+{\left({\sigma}_{sampling}\right)}^2+{\left({\sigma}_{drift}\right)}^2,} $$

where *σ*_*loc*_ is the average localization precision [79], ${\sigma}_{sampling}=2/\sqrt{\alpha }$ where a is the average density of localized single molecules of fluorophores (typically between 4000 and 6000 molecules/μm^2^). Our data generated a theoretical value of average two-dimensional resolution, σ_total_, of 39 nm, resulting in an effective structural resolution of about 90 nm, assuming a normal distribution of the measurement error. In addition, by means of Fourier ring correlation [80], we calculated the lateral resolution in our studies to be approximately 100 nm. Single molecule signals of the DNA dyes, as observed in an optical section through a cell nucleus, are detected on top of a relatively high background. In such cases, the SMLM lateral resolution will be determined mostly by the noise in the background, and not reach as good values as the reported manifold for SMLM [36].

When performing dual-color SMLM imaging, the chromatic shift within cell samples was determined from SMLM experiments of immunostained microtubule standards (double labeling with primary and a mixture of Atto 488-/AlexaFluor 647-coupled secondary antibodies), using the same emission filter set as in the DNA experiments. The shift in the two emission channels of the microtubule data was extracted and then used to correct single fluorophore coordinates from two different detection channels in two-color DNA experiments.

For two-dimensional histogram analysis of stained DNA by SMLM, a square grid of variable width was placed over the cell, and a Matlab function was used to count localized molecules within each square (bin) of the grid. In order to characterize the spatial change in chromatin upon OND, the grid width (bin size) was varied between 10 nm and 500 nm. Subsequently, a histogram of localized single fluorophores per bin was plotted, and the median and quartile values of localizations were determined. This allows us to quantitatively compare the difference in density distribution for control, OND and recovering cells. Nine or more cells were evaluated for each experimental condition. Bins containing less than two localized molecules were discarded from the analysis (DNA-free areas in particular from outside the nucleus).

Analysis of DNA binding dye-free areas in two-dimensioanl SMLM images of Vybrant DyeCycle Violet-stained cells was performed using ImageJ [81] as follows. Reconstructed 8-bit images were subject to histogram stretching in order to cover the entire spectrum of pixel values. Then, a constant threshold for all images analyzed was applied, after which the 8-bit image was converted to a binary image. The area covered by chromatin was calculated by counting the number of pixels in this binary image. Similarly, the total area of the nucleus in the imaging plane was obtained after the chromatin-devoid “holes” were filled (ImageJ function “fill holes”). Then the chromatin free area was calculated by subtracting the chromatin occupied area from total nuclear area.

### SIM of YOYO-1-stained DNA

For SIM imaging, HL-1 cells were treated as described above for SMLM imaging. Cells were stained with YOYO-1 and immediately embedded in Vectashield H-1000 (refractive index 1.45, VectorLabs). We used 488 nm excitation and camera integration times between 200 and 300 ms. A sinusoidal illumination pattern was generated in the focal plane by interference of laser light, resulting in a grid pattern of 280 nm spacing. Three different orientations of the grid with three different phases for each orientation were used, resulting in nine acquired images per two-dimensional slice. The microscope setup and reconstruction software have been described previously [82, 83].

### Supporting data

The data sets supporting the results of this article are available in the Dryad data repository, http://dx.doi.org/10.5061/dryad.d3j00. Available data are: all image sets for the Vybrant Violet/H3K14ac SMLM work (untreated, 9 experiments; OND, 12 experiments; 5 min, 10 experiments; 15 min, 9 experiments; 60 min, 10 experiments; 240 min, 9 experiments). All image sets for the YOYO-1 and EdU SMLM work. All image sets for the DNAseI experiments (untreated, 11 experiments; OND, 11 experiments). All image sets for the H2B-mCherry FRAP study (untreated, 12 experiments; OND, 10 experiments) and for H1.1-GFP FRAP study (euchromatin, 10 experiments; heterochromatin, 11 experiments; OND, 11 experiments). The datasets for flow cytometry: H3, panAc, K9Ac, K27ac, K4me3, K9me3 K27me3 at untreated, 0, 2, 5, 15 30 mins, 1 and 4 hours.

## Additional files

Additional file 1:
**Movie M1.** Generation of a small molecule localization nanograph. (AVI 19512 kb) Additional file 2: Figure S1. The nuclear envelope is not affected by OND, as evaluated by SMLM. **Figure S2** The structure of Lamin B1 is not affected by OND. **Figure S3** Time scales required for YOYO-1 imaging using binding activated localization microscopy (BALM) in the nucleus. **Figure S4** Conventional three-dimensional confocal microscopy is unable to resolve OND-induced chromatin compaction states. **Figure S5** Structured illumination microscopy can detect OND-induced chromatin compaction. **Figure S6** OND reduces chromatin to 120 nm structures. **Figure S7** Fourier ring correlation (FRC) analysis of DNA/SMLM data. **Figure S8** Artifacts in SMLM of DNA analyzed in HL-1 cells subjected to OND. **Figure S9** OND induces a reversible loss of histone H3K9ac as evaluated by immunostaining of cells. **Figure S10** OND induces a reversible loss of histone H3K14ac as evaluated by immunostaining of cells. **Note N1** Artifacts in localization microscopy of chromatin. **Note N2** Factors that limit the observed structural resolution of nuclear structures by SMLM. (DOCX 9506 kb)

## Abbreviations

BrU: bromouridine
BSA: bovine serum albumin
CD: chromatin domain
CDC: chromatin domain cluster
CoA: coenzyme A
EdU: 5-ethynyl-2'-deoxyuridine
FACS: fluorescence-activated cell sorting
FLIM-FRET: fluorescence lifetime imaging microscopy-Förster resonance energy transfer
FRAP: fluorescent recovery after photobleaching
GFP: green fluorescent protein
H: histone
HDAC: histone deacetylase
HIF: hypoxia inducible factor
OND: oxygen and nutrient deprivation
PBS: phosphate-buffered saline
ROI: region of interest
SIM: structured illumination microscopy
SMLM: single molecule localization microscopy
SSC: side-scatter
STD: standard deviation
TF: threshold factor
