# Supplementary material for: A transient ischemic environment induces reversible compaction of chromatin
Source: Genome Biol. 2015 Nov 5;16:246. doi: 10.1186/s13059-015-0802-2 (PMC4635527; doi:10.1186/s13059-015-0802-2)
Supplement: Additional file 2: Figure S1. — The nuclear envelope is not affected by OND, as evaluated by SMLM. Figure S2 The structure of Lamin B1 is not affected by OND. Figure S3 Time scales required for YOYO-1 imaging using binding activated localization microscopy (BALM) in the nucleus. Figure S4 Conventional three-dimensional confocal microscopy is unable to resolve OND-induced chromatin compaction states. Figure S5 Structured illumination microscopy can detect OND-induced chromatin compaction. Figure S6 OND reduces chromatin to 120 nm structures. Figure S7 Fourier ring correlation (FRC) analysis of DNA/SMLM data. Figure S8 Artifacts in SMLM of DNA analyzed in HL-1 cells subjected to OND. Figure S9 OND induces a reversible loss of histone H3K9ac as evaluated by immunostaining of cells. Figure S10 OND induces a reversible loss of histone H3K14ac as evaluated by immunostaining of cells. Note N1 Artifacts in localization microscopy of chromatin. Note N2 Factors that limit the observed structural resolution of nuclear structures by SMLM. (DOCX 9506 kb) [file 13059_2015_802_MOESM2_ESM.docx]

**A transient ischemic environment induces reversible compaction of chromatin**

**Supplemental Information**

Ina Kirmes^1,5^, Aleksander Szczurek^1,5^, Kirti Prakash^1,2^, Iryna Charapitsa^1^, Christina Heiser^1^, Michael Musheev^1^, Florian Schock^1^, Karolina Fornalczyk^1,3^, Dongyu Ma^1,4^, Udo Birk^1^, Christoph Cremer^1,2,6^ and George Reid^1,6^

^1^Institute for Molecular Biology, 55128 Mainz, Germany.

^2^Institute of Pharmacy and Molecular Biotechnology, University of Heidelberg, 69120 Heidelberg, Germany.

^3^Department of Molecular Biophysics, University of Łódź, Poland.

^4^Centre for Biomedicine and Medical Technology Mannheim (CBTM), University of Heidelberg, 68167 Mannheim, Germany

^5^Co-first authors, ^6^co-senior authors.

†To whom correspondence should be addressed. E-mail: C.Cremer@imb-mainz.de, [G.Reid@imb-mainz.de](mailto:G.Reid@imb-mainz.de).

**Supplemental Results**

*OND does not induce a significant change in nuclear volume.* The nuclear volume of HL-1 cells either untreated (90 nuclei) or subject to one hour of OND (76 nuclei) was estimated as described. The nuclear volume of untreated cells had a mean of 757.4 μm^3^, a median of 675.8 μm^3^ and a standard deviation of 242.0 μm^3^. The nuclear volume of OND treated cells had a mean of 745.2 μm^3^, a median of 704.1 μm^3^ and a standard deviation of 191.3 μm^3^. The p-value between both groups using a two-tailed Mann-Whitney-Wilcoxon rank sum test was 0.52, indicating that there was no statistically significant difference between the nuclear volume of untreated and OND treated HL-1 cells.

*OND does not promote invagination of the nuclear envelope.* OND induced compaction of chromatin results in the formation of rod and whorl like structures, which may arise through invagination of the nuclear envelope. This hypothesis was evaluated by determining the structure of Lamin B1, an integral component of the inner nuclear envelope [[1](#_ENREF_1)], by immunohistochemistry of HL-1 cells either untreated or exposed to one hour of OND. As shown in supplemental figure S2, OND does not disturb the structure of the nuclear envelope, as judged by Lamin B1 staining.


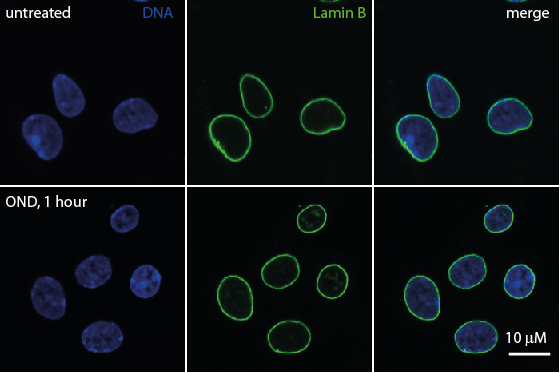


**Supplemental figure S1** *The nuclear envelope is not affected by OND, as evaluated by SMLM.* HL-1 cells, either untreated or subject to OND for 1 hour, were fixed, permeabilised, immunostained for Lamin B1 and their DNA counterstained with Hoechst 33342.


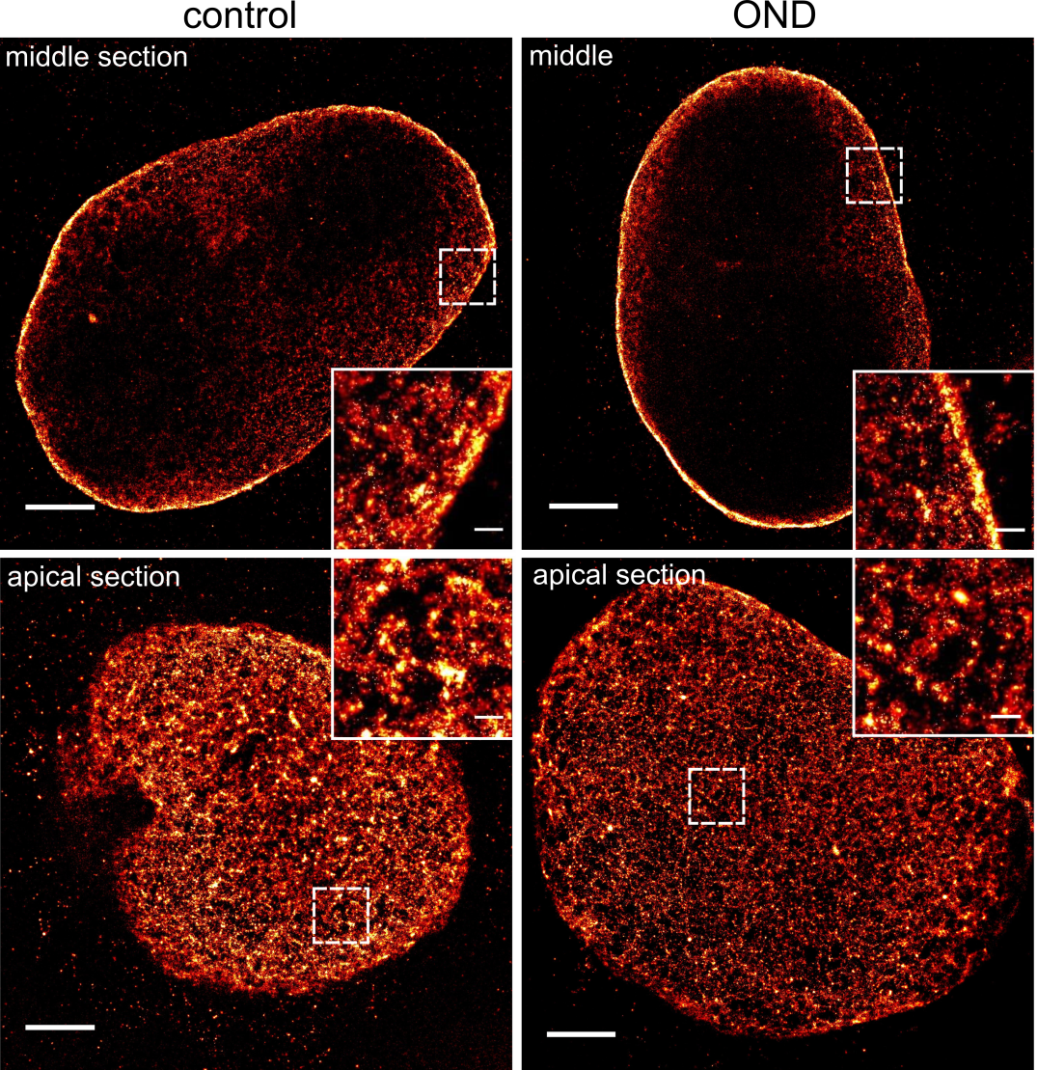


**Supplementary figure S2.** *The structure of Lamin B1 is not affected by OND.* SMLM of Alexa647 immunofluorescently labelled Lamin B1 demonstrates that OND does not induce major structural changes in its distribution, even at the level of tens of nanometers. 2D SMLM images were acquired both at an equatorial part to of the nucleus to demonstrate the association of Lamin B1 with the nuclear envelope and at the bottom of the nucleus to illustrate the Lamin network. Structural features revealed in both cases are in agreement with previous reports [[2](#_ENREF_2)]. Scale bars represent 2 µm in the large micrographs and 200 nm in the insets.

*OND induces a reversible loss of histone acetylation as determined by confocal microscopy.* HL-1 cells, grown on coverslips, were subject to one hour of OND after which they were allowed to recover in normoxic conditions with Claycomb media. Cells were fixed, permeabilized, immunostained with either anti-H3K9ac or H3K14ac and counterstained with Hoechst 33342. All images were generated using identical parameters. As shown in supplemental figures S9 and S10, OND induces a loss of both H3K9ac and H3K14ac, which recovers within 30 minutes following restitution of normoxia and feeding.


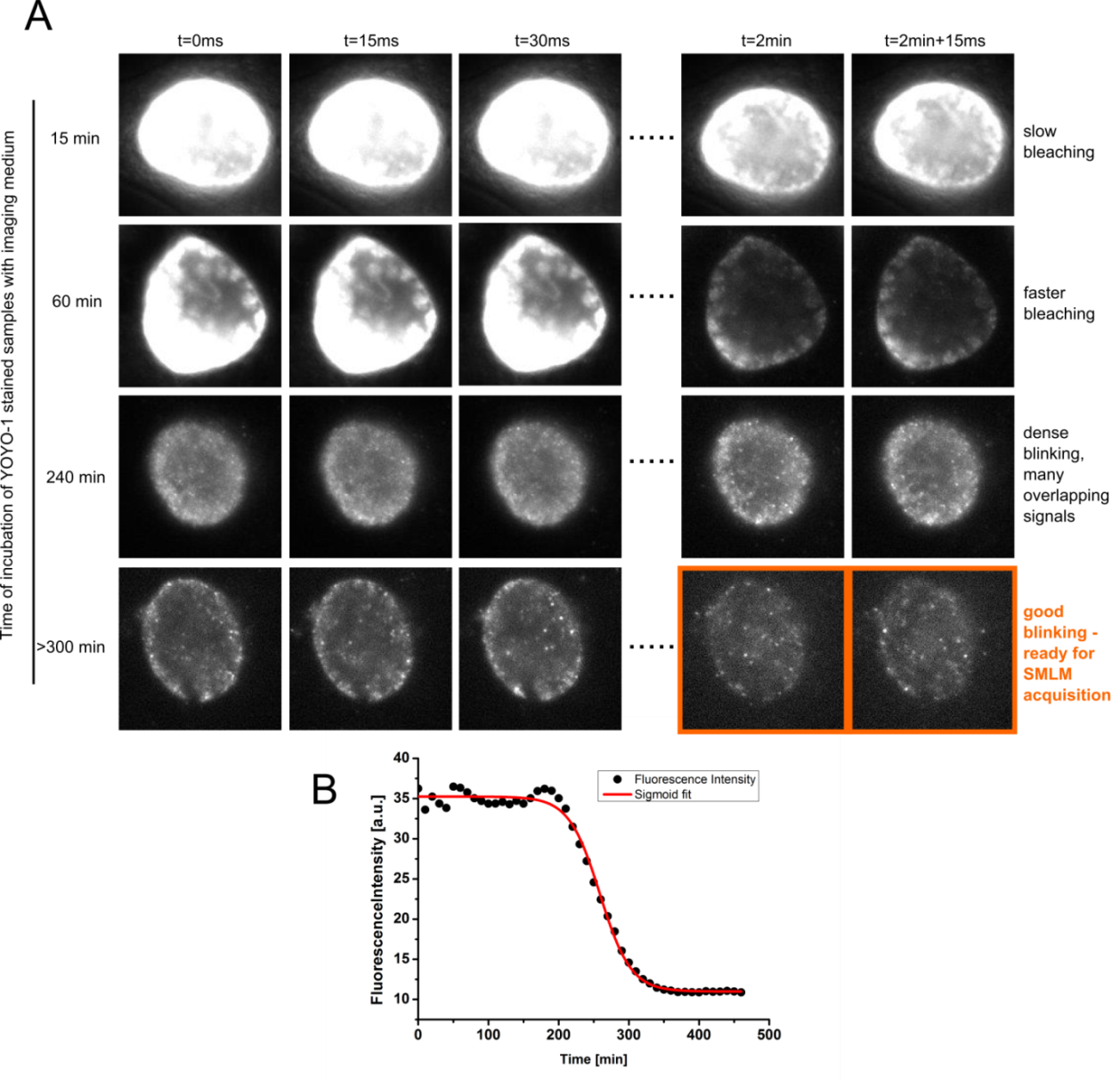


**Supplementary figure S3.** *Time scales required for YOYO-1 imaging using Binding Activated Localization Microscopy (BALM) in the nucleus.* A) A set of SMLM experiments with high intensity 491 nm excitation (for details see Materials and Methods) was performed for different time periods of incubation of YOYO-1 stained cells in imaging buffer (volume of 20 µl), raw images shown. B) time course confocal microscopy demonstrates that decay of signal intensity of YOYO-1 bound to the DNA in time has a sigmoid characteristics. These time-dependent changes are observed in a buffer devoid of oxygen through employing an enzymatic oxygen scavenging system (glucose oxidase and catalase). We anticipate that the basis of unbinding from DNA is a continuously progressing reduction or oxidation of YOYO-1. This is in agreement with previous reports where (un)binding kinetics of YOYO-1 has been enhanced in a presence of reductant and oxidant [[3](#_ENREF_3)]. Additionally, gradual acidification of a buffer due to enzymatic scavenging system might have an influence on chemical properties of YOYO-1 just as red-ox states [[4](#_ENREF_4)]. Preleading cells with YOYO-1 prior to imaging using BALM ensures that YOYO-1 has reached an equilibrium in nuclei; these repetitively bins and unbind to DNA, thereby generating single molecule signals that can be localised. Typically, signals cease to appear in the acquisition after 30 to 40 minutes, suggesting that the YOYO-1 pool within the cell nucleus becomes irreversibly bleached. It is unlikely that the all of the YOYO-1 within the imaging buffer bleaches, as YOYO-1 in solution has a quantum yeild of about 0.001 [[5](#_ENREF_5)]

**
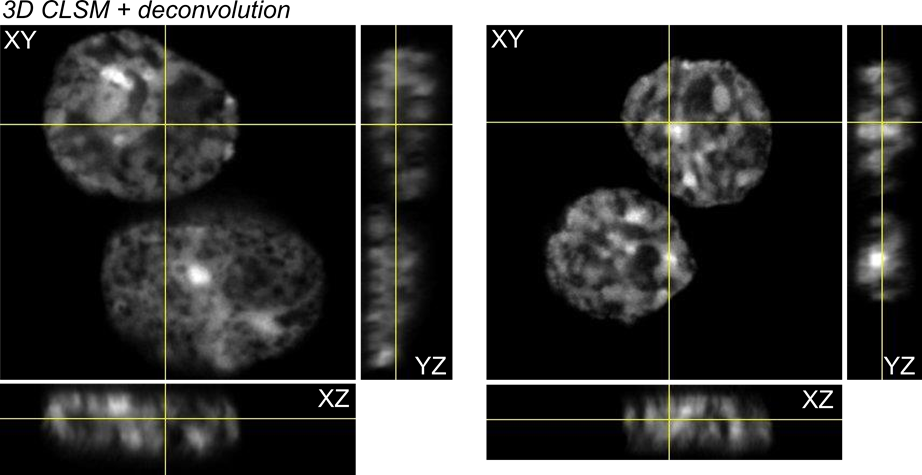
**

**Supplementary figure S4.** *Conventional 3D Confocal microscopy is unable to resolve OND induced chromatin compaction states.* Vybrant dye cycle Violet-stained, OND treated HL-1 cells were subject to evaluation by 3D confocal microscopy using λ_exc_=405 nm, λ_em_=410-480 nm. Two examples following deconvolution and reconstruction are shown, demonstrating that confocal microscopy has insufficient resolving power to reveal the condensation states detectable by means of SMLM.

**Supplementary figure S5.** *Structured illumination microscopy can detect OND induced chromatin compaction.* HL-1 cells were subject to one hour of OND, fixed, permeabilized, treated with RNAse, stained with YOYO-1 and analyzed by structured illumination microscopy. While widefield imaging hints that OND perturbs chromatin structure, SIM clearly reveals chromatin condensation and the development of large chromatin voids. Insets C and D indicate the extent of chromatin compaction induced by OND.


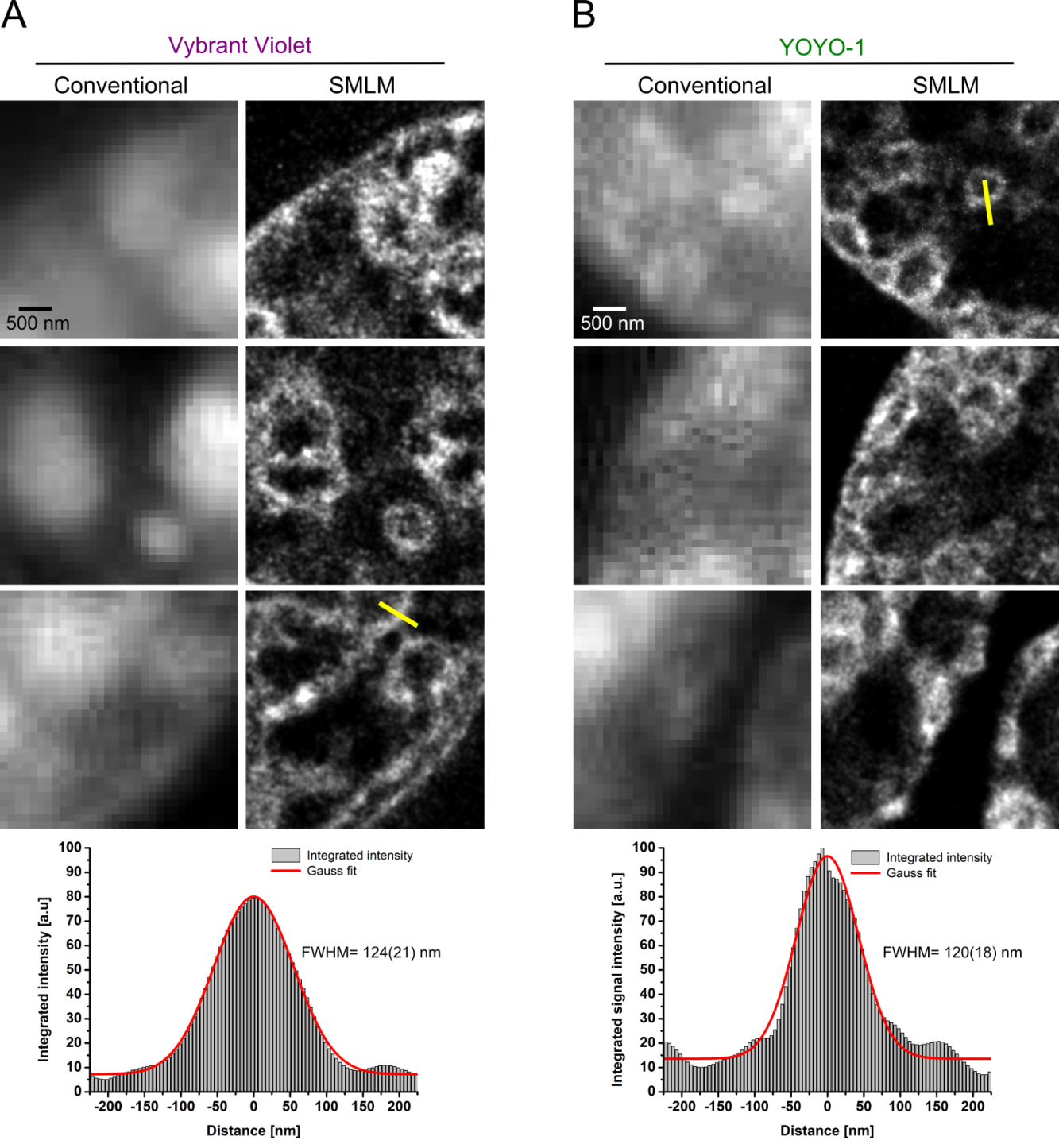
**Supplementary figure S6.** *OND reduces chromatin to 120 nm structures.* Condensation states of chromatin following OND were revealed using single molecule localization microscopy of DNA distribution by means of fluorescent labelling with Vybrant dye cycle Violet (A) or with YOYO-1 (B). Intensity profiles taken in localization images demonstrate the typical thickness of chromatin condensed to the ring-like structures. Average full width at half maximum of these subdiffractional structures amounts to 124 ± 21 nm (Vybrant dye cycle Violet) and 120 ± 18 nm (YOYO-1), n=10. Examples of profiles with gaussian fit are shown. These results are in agreement with the Fourier Radial Corrolation analysis (Supplementary Figure S7 which shows that the size of chromatin structures induced by OND is of the order of 130 nm.


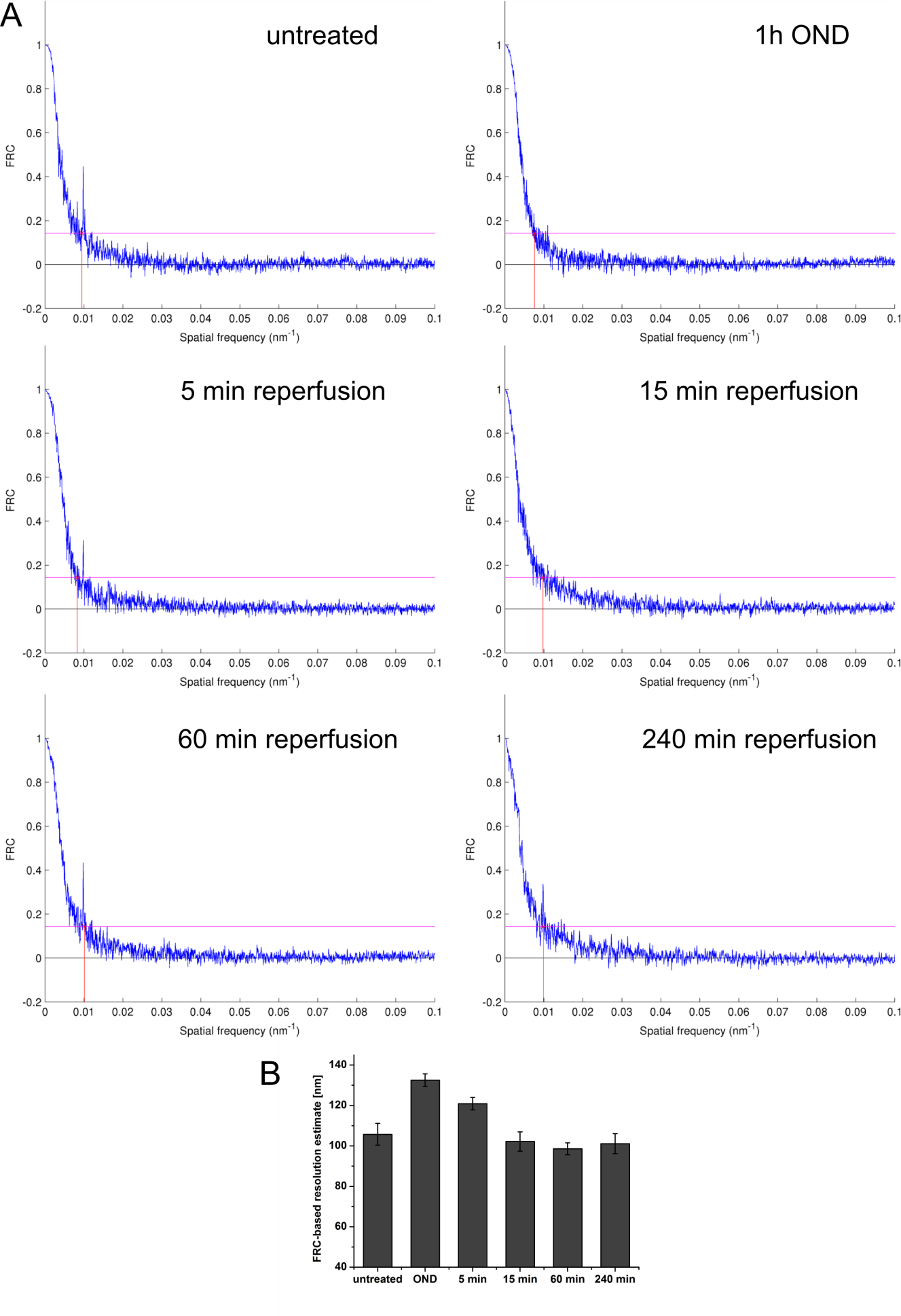


**Supplementary figure S7**. *Fourier Ring Correlation (FRC) analysis of DNA/SMLM data*. A) Representative normalised FRC curves for untreated, OND, and in recovering cells. The red horizontal line designates the 1/7 treshold in accordance with Nieuwenhuizen *et al.* [[6](#_ENREF_6)] of the radially integrated Fourier frequencies. B) Resolution estimates across all experimental conditions based on the treshold determined in A.


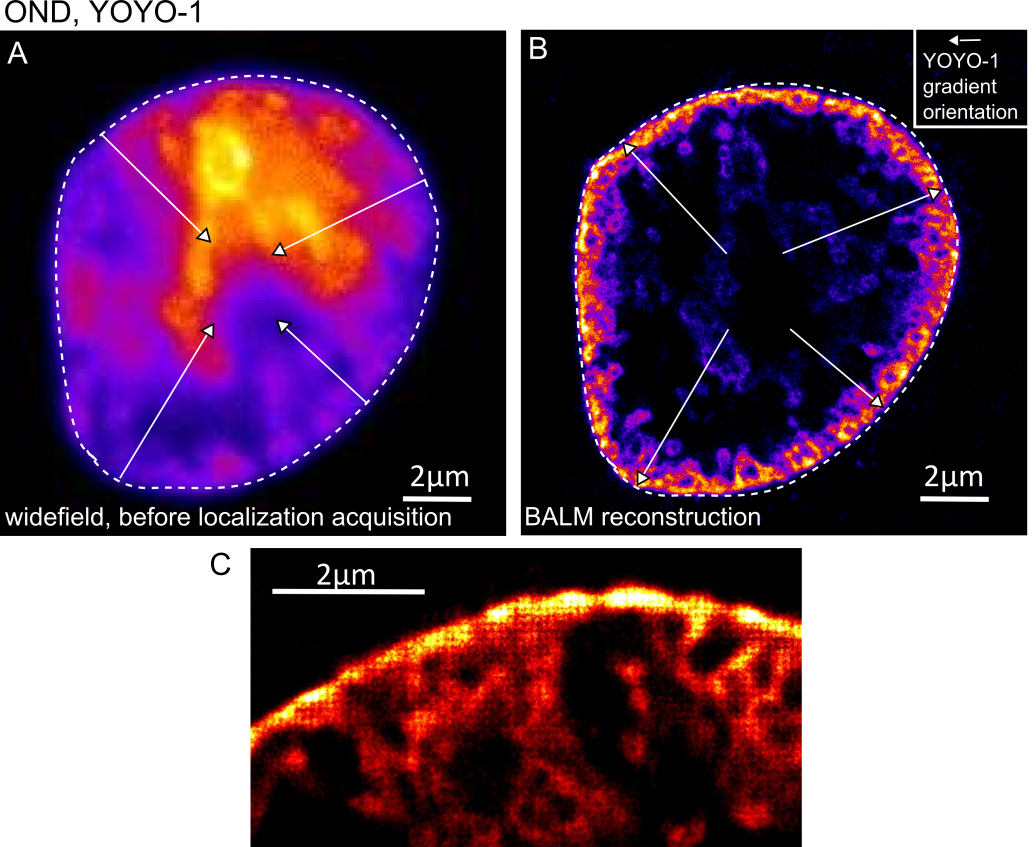


**Supplementary figure S8.** *Artifacts in SMLM of DNA analyzed in OND HL-1 cells*. A) A widefield image of an HL-1 cell nucleus stained with YOYO-1 prior to application of high intensity excitation; a high fluorescent signal inside the nucleus, as compared to the periphery, is apparent. B) The same nucleus reconstructed with single fluorophore molecule signals resulting from the application of high intensity excitation. The single molecule density of heterochromatin at the nuclear periphery greatly exceeds the expected intensity of signal in the central part of the nucleus, as infered from widefield acquisition. C) Grid structures at very densely labeled sites produced as a result of using Center-of-Intensity based SMLM algorithms on data with overlapping single molecule signals. The bias of the SMLM signals to be localized towards the center of the CCD camera pixel was evaluated to result in a deterioration of the localization precision of approximately 4 nm.


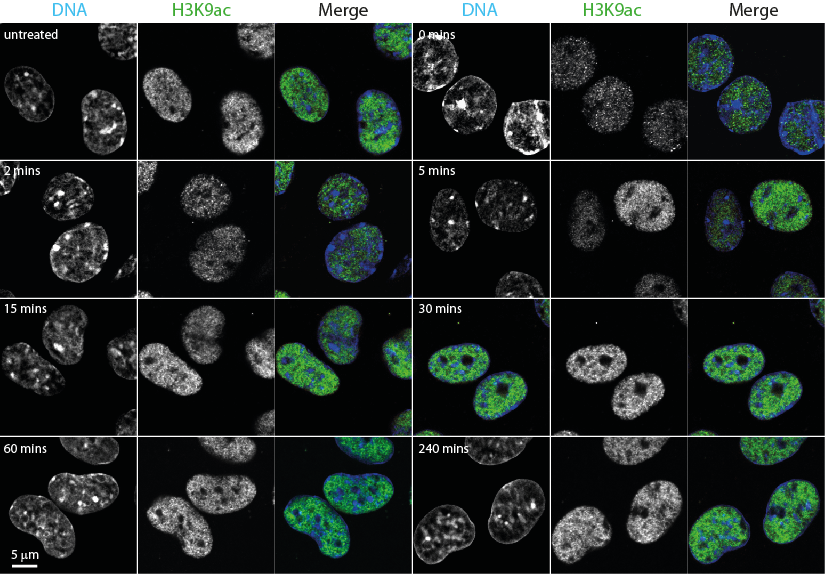


**Supplemental figure S9.** *OND induces a reversible loss of histone H3K9ac as evaluated by immunostaining of cells.*


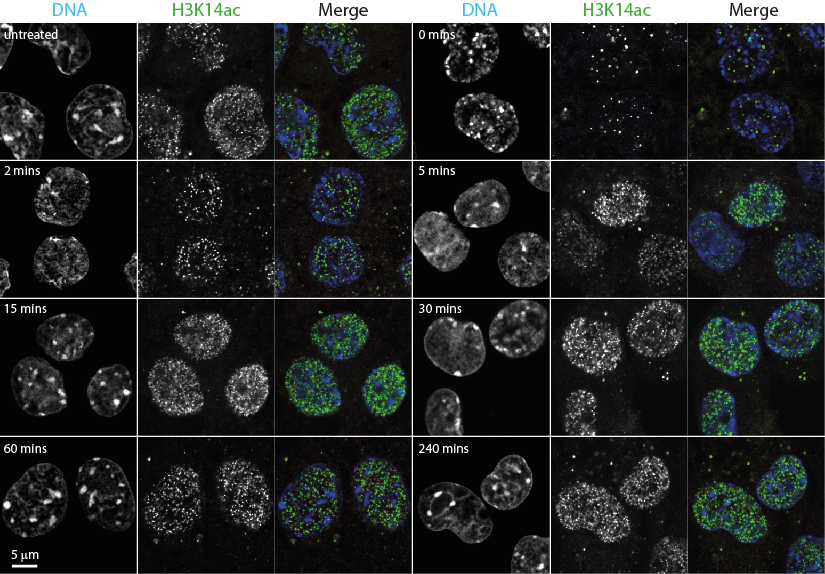


**Supplemental figure S10.** *OND induces a reversible loss of histone H3K14ac as evaluated by immunostaining of cells.*

**Supplementary Note N1:** **Artifacts in localization microscopy of chromatin**

The Binding Activated Localization Microscopy (BALM) method of DNA imaging has been proven to yield excellent quality localization images for easily accessible DNA structures for isolated DNA threads or for a bacterial genome [[3](#_ENREF_3)]. BALM uses environmental conditions that facilitate fast binding-unbinding kinetics of a rapidly diffusing low molecular weight dye (e.g. YOYO-1) that becomes fluorescent upon binding to DNA, with an increase in quantum yield in the order of 800 fold. When a DNA sample is immersed in a low concentration solution of the dye, YOYO-1 transiently binds to DNA and, upon high excitation intensity, emmits ~ 1000 photons, enabling localization using standard PALM/STORM algorithms. Unbound dye is highly mobile and emits very littly fluorescence, and is therefore only visible as weak background in the raw images. Whereas this imaging method is easily applicable to relatively small, accessible DNA structures, BALM may not be optimal when imaging an eukaryotic cell nucleus due to the large size of the cell nucleus. In our hands, the BALM approach as reported previously [[8](#_ENREF_8)] did not yield good results. Therefore, we investigated applicability of the BALM technique in fixed cell samples by staining with YOYO-1, then waiting until it dissociates from DNA into the buffer. At a time period of about 200 min after loading the cells with YOYO-1, this process of dissociation becomes noticeable and then rapidly proceeds. Such approach provides relatively high YOYO-1 concentration even deep inside the nucleus. (supplementary figure S4, unbinding kinetics). In the widefield image, the highest signal is observed in the center of the nucleus. In contrast, in the SMLM/BALM image, only very few YOYO-1 molecules could be detected in the central part of the nucleus. This indicates that the probability to irreversibly bleach YOYO-1 increases with the depth of penetration in nucleus. Under the high excitation intensity necessary for SMLM studies, this leads to formation of a local, intracellular gradient of YOYO-1 capable of fluorescing, with the lowest concentration occuring at the geometric center of the cell nucleus. Consequently, undersampling of chromatin at the centre of the cell nucleus occurs. This introduces variability in the composition of the image, as resolution in localization microscopy is inversely proportional to square root of density of localizations. The influence of fixation on the accessibility of the dye to DNA in the central part of the nucleus has not been studied.

In order to overcome the accessibility issues related to BALM approach, we utilised the photoconvertible dye Vybrant dye cycle Violet and performed SMLM measurements upon permanently bound DNA dye, as we reported previously for DAPI and Hoechst dyes [[9](#_ENREF_9)]. Our SMLM imaging protocol of Vybrant dye cycle Violet delivered reproducible localization density (Figure 2A). In spite of all these advantages, however, the binding mechanism, and possible sequence specificity of Vybrant dye cycle Violet is presently undisclosed which is not the case for YOYO-1, a cyanine dye known to be a DNA intercalator [[10](#_ENREF_10)].

DNA in the eukaryotic cell nucleus is known to be highly compacted, as estimated by the average basepairs per unit volume and in order to investigate chromatin using fluorescence microscopy a high labelling density is required. In localization microscopy, this necessitates a high number of single molecule detections per single aquisition frame and / or the acquisition of a large number of frames. Too high density of active fluorophores inevitably leads to overlapping signals and deterioration of the fine structure reported in SMLM reconstruction. For center-of-gravity-based reconstruction algorithms, a typical non-chaste artifact arising in dense samples is shown in Supplementary figure S8. This artifact is a result of overlapping single molecule signals, and may be removed by filterring out signals with a width larger than mean + 1 standard deviation of the distribution of widths. Here, however, high localization density is crucial and filterring has not been performed. Instead, such data were discarded and not analyzed in this study. Appropriate bleaching ahead of the acquisition reduces the number of single molecule signals per acquired frame to a level where overlapping signals become unlikely.

A further option to image DNA is to use click-chemistry to couple fluorophores to 5-ethynyl-2´-deoxyuridine (EdU) incorporated into DNA during complete DNA replication as presented in figure 2. EdU incorporation however can result in biological artifacts due to toxicity and was shown to upregulate typical DNA repair signaling pathways (e.g. H2AX phosphorylation) as well as initiate cell-cycle arrest prior to mitosis [[11](#_ENREF_11)]. Provoking these cellular responses prior to fixation of the cells certainly has a considerable impact on chromatin structure that is known to change upon DNA damage [[11](#_ENREF_11)]. However, click chemistry to EdU enables coupling easily controllable STORM fluorophores (e.g. AlexaFluor) directly to the DNA, minimizing imaging artifacts [[7](#_ENREF_7)].

**Supplementary Note N2:** **Factors that limit the observed structural resolution of nuclear structures.**

In our measurements of chromatin labeled with DNA binding dyes inside the cell nucleus, we observe a structural resolution of ~90 nm (= 2.35$\sigma_{total}$). In contrast, SMLM measurements of surface structures such as for example membrane bound proteins, typical values for the estimated spatial resolution are in the range of 20 nm. Values of 20 nm or better are not reached in practice inside optically inhomogeneous media, i.e. when focussing through multiple layers of membranes and organelles into the cell nucleus. In this case, the STD (sigma) of the Gaussian-approximated PSF is usually larger than 130nm, and also the noise in the background is often higher due to residual autofluorescence. Reasons for this are as follows:

- We are measuring 3D fixed cell samples, i.e. we observe fluctuating fluorescent background of relatively thick 3D structure of the nucleus due to out-of-focus fluorescent bursts, decreasing the precision of the extracted localization of the single point-like emitters
- We are focusing at a considerable distance from the coverslip (~ 2 - 3um); therefore the width of the fluorescent bursts is broader than the theoretical values for the airy disc.
- We have to deal with a variation of the refractive index inside the cell nucleus (varying from 1.385 for euchromatin to 1.415 for heterochromatin [[12](#_ENREF_12)].
- In addition, the photon yield of Vybrant Dye Cycle Violet is, on average, considerably lower than what has been reported for e.g. Alexa Fluor 647 dyes.

So instead of $\sigma_{loc}$ better than 10 nm, as one would expect from best values for the photon count statistics of Vybrant Dye Cycle Violet fluorophores, we find a value for $\sigma_{loc}$ of at most 25 nm (Formula by Thompson [[13](#_ENREF_13)]; Best values observed in these experiments are ~20 nm). Applying the Mortenson [[14](#_ENREF_14)] formula for the background noise level (standard deviation of ~14 photons) and the photon counts (1500), a upper value of $\sigma_{loc}$ = 37 nm was calculated, corresponding to 87 nm spatial resolution (FWHM of a normal distribution) without any additional drift and at high labelling density. Adding drift and our limited labelling density, we arrive at $\sigma_{total}$ = 48 nm, i.e. 112 nm spatial resolution. The values obtained using the formula presented by Thomspon [[13](#_ENREF_13)] give slightly better values as stated in the supplemental information. Given that it is hardly possible to obtain the theoretical best resolution values when imaging in the cell nucleus, the differences in the various formulas for calculated resolution are of academic interest only, and not relevant to the findings of the paper. Due to the optical aberrations and other difficulties outlined above, also the z-sectioning capability of the SMLM approach presented is lower that what has been reported previously.

2D SMLM reconstructions may typically be confined to represent signals from an axial section covering about 500 - 600 nm [[79](#_ENREF_79)] for bright red fluorophores (e.g. AlexaFluor 647) when a high photon-count in the presence of a low background is obtained. However, in our analyses, we anticipate that the SMLM reconstruction will cover a z-section of less than 500 nm due to the shorter wavelength used for imaging (decreasing the effective axial PSF extent) and relatively low photon count preventing signals slightly out of focus from being detected. The sectioning capability of this method can be seen in supplementary figure S3, where the hollow ring-like DNA structures observed with DNA/SMLM have a lateral diameter of > 500 nm, which we could not resolve using conventional microscopy. Moreover, confocal laser scanning microscopy and structured illumination microscopy having been optimized for best resolution (i.e. lowest possible wavelength) give an indication of the presence of such ring-like structures (supplementary figure S3). In confocal light scanning microscopy (pinhole diameter of 0.5 Airy Units) in combination with deconvolution (supplementary figure S6), condensation into ring structures can occasionally be observed, but is difficult to observe due to limited signal-to-noise ratio.

Due to the considerations outlined above, the images presented appear to be not as crisp as SMLM reconstructions of other types of structures presented previously, either by other groups or by us. However, to the best of our knowledge there is no method for imaging of the nuclear DNA in situ by means of optical methods which was proven (experimentally determined) to give structural resolution better than the values presented by us. We are aware that our method has some drawbacks (in terms of image quality, photon yield, localization accuracy, possible artefacts due to overlapping signals). Furthermore, we show that in spite of these difficulties, the presented technique clearly demonstrates the massive changes in the distribution of nuclear chromatin upon OND treatment.

**Supplementary Movie M1.** *Generating a small molecule localization nanograph.* The steps taken to generate a SMLM image are illustrated for a single cell nucleus that has been exposed to one hour of oxygen and nutrient deprevation then fixed, permeabilized and its DNA labeled with Vybrant DyeCycle Violet. The left-hand window shows the raw data obtained every 50 ms, along with an indication of the laser power used to illuminate the sample. The upper-centre panel indicates the background level of fluorescence, which is subtracted from the raw-data profile to generate the signal above background frame (lower centre). The right-hand window contains the processed localizations, which are integrated to generate a localization map describing chromatin structures at sub-diffractional resolution. Low laser power was used for the first 100 frames to establish background fluorescence, thereafter high laser power was used to bleach most Vybrant DyeCycle Violet molecules. Continued application of high laser power induces a low number of optically isolated molecules of fluorophore to a bright/ON state, as a result of photoswitching. These are filtered to meet criteria of brightness and chastity and their localization accuracy determined, based on the number of photons emitted by the fluorophore. Signals above background are used from frame 200 onwards to contribute to the SMLM map. The next 600 frames (42 seconds) are shown contributing to the SMLM reconstruction. Thereafter, the final reconstructed image, consisting of 25,000 frames (imaging for 28 minutes and 44 seconds) is shown.

**Glossary**

*Adenosine Triphosphate (ATP)* Coenzyme used as an energy carrier in cellular metabolism. The polyphosphate tail of ATP associates with intracellular divalent cations, principally Mg^2+^, and with polyamines, such as spermine and spermidine, which are liberated upon depletion of ATP.

*Binding activated localization microscopy (BALM)* – In this technique, fluorescent molecules that are typically invisible to the detector while in solution become visible (emit fluorescence in the detection range) upon binding to the structure of interest; for example YOYO-1, which undergoes over a thousand fold increase in green fluorescence when bound to DNA). Typically, the fluorophore remains bound to the structure for a few up to tens of ms, during which, under exposure to high intensity light, results in individual bound molecules emitting hundreds to thousands of photons. Data collected over extended time periods enables sufficient sampling of the structure based on localizing single probe signals (see also *single molecule localization*).

*Cardiomyocyte* Cardiac muscle cells with a high mitochondrial density, which allows them to produce adenosine triphosphate (ATP) quickly, making them highly resistant to fatigue. This study employed the immortal murine cardiomyocyte cell line HL-1, generated by William Claycomb [[15](#_ENREF_15)], as an appropriate model to investigate cardiac ischemia.

*Chromatin* is a complex of DNA, protein and RNA, consisting primarily of 147 basepairs of DNA wrapped 1.67 times around nucleosomes in a left-handed superhelix. Nucleosomes then further associate to achieve higher orders of compaction. Two functions of chromatin are to organise DNA into a compact structure and to regulate transcriptional activity. The regulatory platform of transcription is chromatin.

*Chromosome territory* Chromosomes occupy specific regions, known as chromosome territories, within the nucleus. Chromosome territories are exclusive for each chromosome with homologous chromosomes present in separate locations. Transcriptionally active regions of chromosomes are located at the edge of chromosome territories, with inactive and condensed chromatin present positioned within chromosome territories.

*EdU* EdU (5-ethynyl-2'-deoxyuridine) is a nucleoside analogue of thymidine that can be used to uniformly label DNA through incorporation during DNA synthesis. Labelled DNA can then be detected using rapid, highly specific “click-chemistry”, a copper-catalyzed covalent reaction between an azide and an alkyne that attaches a fluorophore to labelled DNA.

*Epigenetic* Historically, epigenetics was restricted to mitotically and/or meiotically heritable changes in gene function, beyond those resulting from DNA sequence variation. This has been broadened to "the structural adaptation of chromosomal regions so as to register, signal or perpetuate altered activity states” [[16](#_ENREF_16)]. This definition includes transient modifications associated with DNA repair or cell-cycle phases as well as stable changes maintained across multiple cell generations.

*Fluorophore blinking* is the repetitive emission of > 10^3^ photons under excitation illumination by a single, optically isolated molecule of fluorophore upon arrival to the bright/ON state, as a result of photoswitching or of stochastic circulation between states.

*Fluorophore dark/OFF state* - A physicochemical state of a fluorescent probe in which it is not "visible" to the detector, as it is not capable of emitting light in the emission band of interest. This may arise from a transient inability to absorb the excitation light. In standard microscopy, such bleaching contributes to an unwanted loss of fluorescence signal. However, in SMLM, a high abundance of bleached probes enables discrimination of single fluorescing (blinking) molecules localised within the observation volume during imaging. The formation of the 'dark'/'OFF' state might arise from the reduction, oxidation or transient chemical reaction of a fluorophore. In consequence a 'bright'/'ON' state can be induced through photoconversion, a shift in absorption/emission spectra; photoactivation, a gain in fluorescent properties and by stochastic circulation.

*Histones* are small, highly alkaline proteins found in eukaryotic cell nuclei that package and order DNA into structural units known as nucleosomes. They are the major protein components of chromatin, acting as spools around which DNA winds. Five major families of histones exist, H1/H5, H2A, H2B, H3 and H4. Histones H2A, H2B, H3 and H4 constitute core histones with two H2A-H2B dimers and a H3-H4 tetramer assembling into the core histone, while histones H1 and H5 are linker histones. In addition to their structural role, histones have a major role in gene regulation through post-translational modifications, particularly on their unstructured N-terminal tail. In this study, we evaluated a number of post-translational modifications of lysine residues of histone H3 with regard to their behaviour upon OND induced chromatin condensation. Specifically, acetylation of lysine residues of histones neutralises their positive charge, reduces the strength of interaction between the nucleosome and DNA and is associated with transcriptional activation. Methylation of lysine residues can permit (for example H3K4me3, as reported in this study) or repress (for example H3K9me3 and H3K27me3, as reported in this study) transcriptional activation.

*Hypoglycemia* refers to low blood glucose levels. In the context of this study, ischemia results in the affected tissue having a restricted blood supply, and in consequence, insufficient glucose to maintain oxidative metabolism. Hypoglycemia contributes to the depletion of cellular ATP levels that occur upon an insufficient supply of blood.

*Interchromosomal space* is the functional nuclear compartment located between chromosome territories. The intercromosomal space, while chromatin sparse, contains nuclear bodies enriched in gene expression and RNA processing machinery. Chromatin positioning is restricted to regulate access of transcriptional units to the interchromosomal space. OND induced chromatin compaction, while increasing the volume of the interchromosomal space, reduces the area of potential interaction between chromatin and the interchromosomal space.

*Ischemia* is a restriction in blood supply to tissue, resulting in a shortage of oxygen (hypoxia) and of glucose (hypoglycaemia). Cells affected by ischemia predominatly generate their energy through glycolysis, resulting in the local production of lactic acid, which induces a reduction in pH within ischemic tissue.

*Nyquist sampling theorem* describes the relationship between a continuous signal, in our case total chromatin, and a digital, or discrete, derivation of the signal, in our case SMLM of fluorescent dyes bound to DNA. The Nyquist theorem describes a sufficient extent of sampling that captures all the information within the continuous signal. In our manuscript, we observed that in a binning approach, chromatin structures in bins with edges from 20 to 280 nm were variant between untreated and OND treated cells. The Nyquist sampling theorem dictates that the actual size of the structures involved must be twice the size of the bin dimension we evaluated.

*Oxygen and Nutrient Deprivation (OND)* in our studies, we mimicked the environmental effects of ischemia by culturing cells in a defined hypoxic environment in buffered media devoid of an energy source and that blocked glycolysis with the glucose analogue deoxglucose.

*Photoswitching* is a photoinduced change to the absorption or emission characteristics of a fluorophore that results in the acquisition or loss of fluorescent properties in the emission range of detection. This results in continuous interconversion between the 'dark'/'OFF' state and the 'bright'/'ON' state.

*Polyamines* are organic compounds containing two or more primary amine groups. In cells, the main polyamines are putrescine, spermidine and spermine. They are primarily sequestered on the cellular polyphosphate pool, mainly on ATP. In our work we propose that ATP depletion results in liberation of the polyamine pool, which relocates to DNA provoking histone displacement and chromatin condensation.

*Single molecule localization* is an process in which the estimated position of single fluorophore signals is derived from raw multiframe acquisitions. This is often attained using point spread function fitting or by centre of gravity approaches, which enables localization of single molecules of fluorophores with subpixel accuracy, often down to a few nanometers.

*Single molecule localization microsocpy (SMLM)* is an imaging technique based on fluorescent widefield microscopy. It is dependent on the induction of a 'dark'/'OFF' state in the vast majority of fluorescent probes within the observation volume, while detecting the minor visible fluorophores over seconds to minutes using a sensitive CCD camera. This is routinely achieved through provision of a specific chemical environment or by utilising photoswitchable fluorophores. Cycling of the fluorophores between a 'dark'/'OFF' state and a 'bright'/'ON' state (see *photoswitching*) enables acquisition of over 10^6^ molecules. After localization (see *single molecule localization*) their estimated positions are integrated into a joint localization map that can determine structural features with a resolution down to 20 nm.

*Structured Illumination Microscopy* is is a widefield fluorescence microscopy technique employing an illumination pattern in order to obtain higher resolution images. Instead of a single image per focus position, multiple images are acquired with different orientations and positions of the illumination pattern. Using sophisticated computer algorithms, these images are then combined to form a superresolution image.

*Vybrant DyeCycle Violet* is a fluorescent, cell permeable DNA binding dye which undergoes reversible photoswitching and that can be utilized for SMLM based on blinking, with individual fluorophores emitting up to 2x103 photons per cycle. The structure and mode of binding of Vybrant DyeCycle Violet has not been disclosed.

*YOYO-1* is a cell-impermeable, green, fluorescent, cyanine dye that can be utilized for SMLM based on blinking. While YOYO-1 has a preference for DNA, it also binds with lower affinity to RNA, necessitating RNAse treatment of cells prior to SMLM analysis.

*Z-score* is the number of standard deviations an observation is from the mean of a population. A positive z-score indicates that the observation is greater than the population mean, with a negative z-score indicating that the observation is less than the mean. Z-scores were used in this manuscript to indicate the extent of differences between observations based upon tens of thousands of datapoints.

References

1. Schuler E, Lin F, Worman HJ: **Characterization of the human gene encoding LBR, an integral protein of the nuclear envelope inner membrane.** *J Biol Chem* 1994, **269:**11312-11317.

2. Schermelleh L, Carlton PM, Haase S, Shao L, Winoto L, Kner P, Burke B, Cardoso MC, Agard DA, Gustafsson MG, et al: **Subdiffraction multicolor imaging of the nuclear periphery with 3D structured illumination microscopy.** *Science* 2008, **320:**1332-1336.

3. Schoen I, Ries J, Klotzsch E, Ewers H, Vogel V: **Binding-activated localization microscopy of DNA structures.** *Nano Lett* 2011, **11:**4008-4011.

4. Shi X, Lim J, Ha T: **Acidification of the oxygen scavenging system in single-molecule fluorescence studies: in situ sensing with a ratiometric dual-emission probe.** *Anal Chem* 2010, **82:**6132-6138.

5. Cosa G, Focsaneanu KS, McLean JR, McNamee JP, Scaiano JC: **Photophysical properties of fluorescent DNA-dyes bound to single- and double-stranded DNA in aqueous buffered solution.** *Photochem Photobiol* 2001, **73:**585-599.

6. Nieuwenhuizen RP, Lidke KA, Bates M, Puig DL, Grunwald D, Stallinga S, Rieger B: **Measuring image resolution in optical nanoscopy.** *Nat Methods* 2013, **10:**557-562.

7. Zessin PJ, Finan K, Heilemann M: **Super-resolution fluorescence imaging of chromosomal DNA.** *J Struct Biol* 2012, **177:**344-348.

8. Heilemann M, van de Linde S, Mukherjee A, Sauer M: **Super-resolution imaging with small organic fluorophores.** *Angew Chem Int Ed Engl* 2009, **48:**6903-6908.

9. Szczurek AT, Prakash K, Lee HK, Zurek-Biesiada DJ, Best G, Hagmann M, Dobrucki JW, Cremer C, Birk U: **Single molecule localization microscopy of the distribution of chromatin using Hoechst and DAPI fluorescent probes.** *Nucleus* 2014, **5:**331-340.

10. Rye HS, Yue S, Wemmer DE, Quesada MA, Haugland RP, Mathies RA, Glazer AN: **Stable fluorescent complexes of double-stranded DNA with bis-intercalating asymmetric cyanine dyes: properties and applications.** *Nucleic Acids Res* 1992, **20:**2803-2812.

11. Zhao H, Halicka HD, Li J, Biela E, Berniak K, Dobrucki J, Darzynkiewicz Z: **DNA damage signaling, impairment of cell cycle progression, and apoptosis triggered by 5-ethynyl-2'-deoxyuridine incorporated into DNA.** *Cytometry A* 2013, **83:**979-988.

12. Solovei I, Kreysing M, Lanctot C, Kosem S, Peichl L, Cremer T, Guck J, Joffe B: **Nuclear architecture of rod photoreceptor cells adapts to vision in mammalian evolution.** *Cell* 2009, **137:**356-368.

13. Thompson RE, Larson DR, Webb WW: **Precise nanometer localization analysis for individual fluorescent probes.** *Biophys J* 2002, **82:**2775-2783.

14. Mortensen KI, Churchman LS, Spudich JA, Flyvbjerg H: **Optimized localization analysis for single-molecule tracking and super-resolution microscopy.** *Nat Methods* 2010, **7:**377-381.

15. Claycomb WC, Lanson NA, Stallworth BS, Egeland DB, Delcarpio JB, Bahinski A, Izzo NJ: **HL-1 cells: A cardiac muscle cell line that contracts and retains phenotypic characteristics of the adult cardiomyocyte.** *Proc Natl Acad Sci U S A* 1998, **95:**2979-2984.

16. Bird A: **Perceptions of epigenetics.** *Nature* 2007, **447:**396-398.
